# LightGBM integration with modified data balancing and whale optimization algorithm for rock mass classification

**DOI:** 10.1038/s41598-024-73742-9

**Published:** 2024-10-03

**Authors:** Long Li

**Affiliations:** School of Management Science and Engineering, Shandong Technology and Business University, Yantai, 264005 Shandong China

**Keywords:** Data balancing algorithm, Improved whale optimization algorithm, Rock mass classification, Light gradient boosting machine, Civil engineering, Computer science

## Abstract

The accurate prediction of uneven rock mass classes is crucial for intelligent operation in tunnel-boring machine (TBM) tunneling. However, the classification of rock masses presents significant challenges due to the variability and complexity of geological conditions. To address these challenges, this study introduces an innovative predictive model combining the improved EWOA (IEWOA) and the light gradient boosting machine (LightGBM). The proposed IEWOA algorithm incorporates a novel parameter *l* for more effective position updates during the exploration stage and utilizes sine functions during the exploitation stage to optimize the search process. Additionally, the model integrates a minority class technique enhanced with a random walk strategy (MCT-RW) to extend the boundaries of minority classes, such as Classes II, IV, and V. This approach significantly improves the recall and F_1_-score for these rock mass classes. The proposed methodology was rigorously evaluated against other predictive algorithms, demonstrating superior performance with an accuracy of 94.74%. This innovative model not only enhances the accuracy of rock mass classification but also contributes significantly to the intelligent and efficient construction of TBM tunnels, providing a robust solution to one of the key challenges in underground engineering.

## Introduction

In the geological classification system used in China, rock masses are systematically categorized into five classes ranging from Class I to Class V^1–3^. Real-time assessment of rock mass classes in TBM tunneling is critical for ensuring safety and efficiency. However, it presents significant challenges due to complex and variable geological conditions. For example, factors such as crack geometries, mechanical properties, and water-rock interactions complicate the prediction of rock mass behavior^4–7^. This complexity has driven significant research interest in creating predictive models. These models use advanced machine learning algorithms to handle the geological challenges. Despite advances, most predictive models rely heavily on data derived from TBM operations^8–10^ and often face critical limitations, such as class imbalance. Class III rock masses dominate TBM tunneling data, representing over half of the total. Minority classes like Class V are underrepresented, causing prediction inaccuracies of about 69%^11^. Therefore, identifying and addressing these gaps is vital to improve predictive performance and ensure safer tunneling operations. Existing research often fails to handle data imbalance effectively. It also lacks adaptability to complex geological environments, revealing a major gap in current methodologies. Traditional models often struggle with minority class predictions because they focus on minimizing overall errors. This approach frequently leads to suboptimal outcomes for smaller classes like Classes II and V. Addressing these shortcomings is crucial to enhance model reliability and practical applicability in TBM projects, which is the primary focus of this study.

This research aims to develop innovative data-balancing algorithms and improve predictive models. The goal is to enhance the accuracy of rock mass classification in TBM tunneling, especially for underrepresented classes. This work aims to overcome the limitations of traditional approaches by introducing new methodologies that can better handle data skewness and complex geological conditions.

Recent research in the mining and tunneling industries has focused on integrating advanced computational techniques to address various operational challenges^11,12^, particularly those related to safety, environmental impact, and predictive performance. For example, the application of fuzzy inference systems combined with genetic algorithms and pattern search has been used to predict roof fall rates in underground coal mines, providing a robust approach to enhancing safety through data-driven decision-making^13^. Building on foundational studies, recent work has explored statistical and AI methods for predicting performance in hard rock TBM operations. These studies show the potential of AI-driven models in optimizing tunneling processes^14^. Optimization algorithms have been used to predict the rate of penetration in petroleum drilling. This application highlights the value of advanced modeling techniques in improving drilling efficiency and decision-making accuracy^15^. The integration of intelligent decision-making strategies is also becoming increasingly prevalent in subsurface engineering environments. For instance, strategies have been developed to predict fire intensity, enhancing safety management in hazardous conditions^16^. Machine learning approaches like T-SNE, k-means clustering, and XGBoost have been used to model short-term rock burst events. These methods significantly improve the stability assessments of subsurface structures^17^. Decision intelligence-based predictive modeling has been used for hard rock pillar stability and air quality index assessments. These applications show the adaptability and effectiveness of these approaches in mining and related industries^18,19^. This growing body of work highlights the trend towards integrating advanced predictive and optimization algorithms, directly addressing the critical challenges identified in TBM tunneling (such as safety). However, existing solutions often do not sufficiently address the unique data imbalance challenges present in TBM operations, further emphasizing the need for tailored approaches.

Numerous scholars have explored various approaches to address these challenges, including feature selection, clustering, algorithm selection, and data balancing. Given the complex, high-dimensional data generated during TBM tunneling, researchers have adopted data-driven strategies. These strategies help identify effective combinations of features. This effort is directed towards reducing the ramifications of data skewness^20^. Within the domain of clustering, a range of sophisticated techniques, including the k-means + + algorithm and spectral clustering, have been employed to reconceptualize the original data structure. These approaches have been used to restructure the four existing classes of rock masses into four or five distinct classes^21,22^. However, research on reclassifying rock mass classes overlooks the crucial need for interpretability in practical engineering applications of the model.

Research has increasingly focused on utilizing diverse algorithms to enhance the accuracy of predicting rock mass classes. These methodologies range from the regression tree^23^ and adaboost-integrated regression tree^24^ to BP neural networks^25^, CatBoost enhanced with sequential model-based optimization^26^, support vector machines (SVMs)^27^, and xtreme gradient boosting (XGBoost) and the light gradient boosting machine (LightGBM)^28^. Although most studies have focused on the utilization of these advanced algorithms, the issue of data imbalance remains an unresolved challenge. Acknowledging these factors, some researchers have adopted the synthetic minority oversampling technique (SMOTE)^29^ to balance datasets, focusing particularly on improving the performance of samples from minority classes^30^. Many advancements in SMOTE have been made to address various complex data scenarios. Examples include ADASYN^31^, Borderline-SMOTE^32^, SMOTE-ENN^33^, k-means-SMOTE^34^, and Random-SMOTE^35^.

SMOTE was developed to mitigate data imbalance in TBM-related fields. However, it inadvertently introduces additional data noise, complicating the classification outcomes. This issue becomes especially problematic when distinguishing between rock mass classes. Data attributes like the penetration rate often display similarities, particularly between Classes II and III. The excessive generation of samples at the boundaries of Classes II and III by SMOTE paradoxically exacerbates this challenge, causing the model more difficulty in differentiating between these closely aligned classes. The random oversampling technique, which involves selecting and replicating samples from the minority class, is an alternative strategy. This method significantly addresses pronounced skewness in rock mass class data. However, this approach introduces a risk of overfitting because of its random replication process^36^.

The minority class technique (MCT) clones each instance of a minority class based on its similarity to the mode. This approach enhances the model performance by altering the class distribution within the training set^37^. Therefore, the MCT is suitable for addressing the prediction of rock mass classes. Although the MCT can mitigate the effects of severe data skewness in rock mass class data, it fundamentally relies on replicating the original data and does not extend the boundaries of minority rock classes.

To address the shortcomings of the existing research, this study introduces an innovative data-balancing algorithm. The proposed methodology aims to enhance the accuracy of rock mass class prediction. It does so by modifying the class distribution in the training set and extending the boundaries of the minority class. Initially, the MCT was refined using the Manhattan distance to improve the generalization capacity of the replicated data. Furthermore, we incorporated a random walk (RW) strategy^38^ to expand the boundaries of minority-class rock masses. The central concept of the RW strategy involves the creation of new values by adding a small random disturbance to the original attribute values (feature values). The disturbance was calculated based on the mean and standard deviation (Std) of the replicated samples. This dual approach addresses the major challenges of data imbalance and classification noise, significantly improving the model’s performance in TBM tunneling applications.

Recent studies have demonstrated the effectiveness of LightGBM for the accurate prediction of rock mass classes^20,28^. LightGBM tackles dataset imbalance by adjusting the sample weights, enhancing the focus on minority rock mass classes, and boosting the prediction accuracy. The gradient boosting algorithm further corrects misclassifications in these classes, which is crucial for imbalanced datasets. However, hyperparameter configuration is challenging because varying combinations yield significantly diverse outcomes. Recently, engineers have exhibited considerable interest in applying metaheuristic algorithms for the minimization of cost functions to achieve optimal solutions. The strengths of these algorithms include their ability to manage nonlinear problems and avoid falling into local optima. Among these are algorithms such as particle swarm optimization^39^, the gray wolf optimizer (GWO)^40,41^, the whale optimization algorithm (WOA)^42^, and Harris hawk optimization^43^. This situation positions metaheuristic algorithms as promising approaches for the hyperparameter optimization of LightGBM models, offering a pathway to refine and enhance their predictive performance.

Metaheuristic algorithms, which are proficient in tackling optimization challenges, may not always be ideally suited for a broad spectrum of diverse problem scenarios. This limitation has led to the development of various specialized and modified algorithms^44^. Reflecting this trend, the WOA, known for its simple yet effective search mechanism, is a prime example. By leveraging the foundational principles of the WOA, researchers have developed various enhanced iterations, including the LWOA^45^, enhanced WOA (EWOA)^46^, and IWOSSA^47^. Acknowledging the specific nature of balanced data for surrounding rock classes characterized by substantial size and high dimensionality, we introduced an optimized WOA variant tailored for the prediction of surrounding rock classes. Specifically, the modification enhances the parameter *l* and hunting behavior of whales (affecting the exploration and exploitation stages) to strengthen the global search capabilities.

The main findings of this study are as follows:

1. The proposed MCT-RW algorithm innovatively achieves the equalization of rock mass classes through a dual-aspect approach.
2. An improved WOA is introduced that balances exploration and exploitation.
3. A novel improved EWOA (IEWOA)-MCR-RW algorithm with LightGBM was established and validated using TBM project data.
4. The results show that the proposed algorithm can improve the performance of rock mass class prediction.

The remainder of this paper is organized as follows. “Methodology” section details the principles of MCT-RW, IEOA, LightGBM, and the evaluation metrics employed. “Framework of the IEWOA-MCT-RW-LightGBM forecast model” section describes the framework adopted to predict rock mass classes in this study. “Case study data” section elaborates on the case data and feature selection process. “Model establishment and validation” section describes the validation process of the IEWOA, including the testing and validation procedures for the rock mass class prediction model. Section 6 presents a detailed discussion of the comparative results of SMOTE-based algorithms and various models, addresses the limitations of this work, and outlines future research directions. “Discussion” section concludes the paper by summarizing the key findings and contributions of this study.

## Methodology

### MCT-RWO algorithm

#### MCT

This section introduces the MCT. The core concept of the MCT is that, in addressing class imbalance issues, instances internal to the class are more reliable than boundary instances. The algorithm clones instances based on their similarity to the mode of the minority class^37^, thereby altering the class distribution. Instances with higher similarity, closer to the class center, are cloned more frequently than boundary instances. However, this method of computing similarity is more suitable for discrete data types. Consequently, Kovács^48^ modified the MCT by replacing the mode with the median and similarity with the Euclidean distance. Normalization was then performed to handle continuous numerical types more effectively.

The summary of the MCT provided by Kovács^48^ is as follows:

1. Establishing simplex sampling: The use of the k-nearest neighbor algorithm to identify the neighbors of each sample within a minority class facilitates simplex sampling, thereby establishing the local structure of the data. These simplex samples are employed in the subsequent sample generation process. Notably, variations in the number of neighbors (k-value) do not significantly affect the quantity of the ultimately generated samples.
2. Calculation of the median: The median of the minority class samples is computed and serves as a reference point to assess the position of other minority class samples relative to the data center.
3. Distance calculation: The Euclidean distance is used to measure the distance from each minority class sample to the median. This distance metric provides a relative measure of the proximity of each sample to the center point.
4. Distance normalization: The obtained distances are normalized to create a distribution that represents the relative importance of each minority class sample. This normalization ensures that the distance metrics are balanced in terms of their influence on the generation of new samples. Sample points with higher weights are closer to the median and considered more important.
5. Sample generation: New samples are generated within the geometric structures formed by the minority class samples and their neighbors according to the weight distribution. This process aims to increase the number of minority class samples while maintaining the data characteristics.

The choice of the median over the mode as a measure of central tendency effectively reduces the impact of TBM body vibrations on data analysis stability. The median is inherently more robust against extreme values and skewed distributions, providing a more consistent measure in environments with mechanical vibrations^48^. The Manhattan distance, which measures absolute differences between points, is less affected by minor data fluctuations. In contrast, the Euclidean distance amplifies variations through squaring. This makes the Manhattan distance a more suitable metric for scenarios involving operational variability in TBM processes. The Manhattan distance between points *U* (*u*_1_, *u*_2_) and *V* (*v*_1_, *v*_2_) is calculated as follows:

1 $$S(U,V)=|{u_1} - {v_1}|+|{u_2} - {v_2}|.$$

Given that the MCT merely replicates the original samples without extending the boundaries between different classes, we introduced an RW strategy in this study. The subsequent sections explore the theoretical foundations of the RW strategy.

#### RW strategy

In the exploration of multi-attribute datasets, for each attribute *b*_*i*_ in the minority class samples, its mean and Std are calculated and denoted as *µ*_*i*_ and *σ*_*i*_, respectively. Each attribute can be regarded as a random variable, and each data point derived from this attribute is considered as a sample value of that random variable. For the attribute *b*_*i*_, its actual mean and Std are represented as *µ*_*i*_′ and *σ*_*i*_′, respectively. If the number of instances *n* in the minority class approaches infinity, then the following holds^38^:

2 $$\frac{{{u_i} - {{u^{\prime}}_i}}}{{{{\sigma ^{\prime}}_i}/\sqrt n }} \to N(0,1).$$

Assuming that each attribute is independent, Eq. (2) is applicable to all attributes^38^. Given that Eq. (2) holds true as the sample size *n* tends towards infinity, the parameters of an unknown distribution can be approximated. Consequently, the following holds true:

3 $${u^{\prime}_i}={u_i} - s \times \frac{{{{\sigma ^{\prime}}_i}}}{{\sqrt n }}$$

where *s* represents a sample drawn from the normal distribution *N*(0,1). By replacing the mean with an attribute value, given the value of attribute *b*, *b*_*i*_(*j*), the synthetic value *b*_*i*_(*j*) for *b*_*i*_ can be generated:

4 $${b_i}^{\prime }(j)={b_i}(j) - {s_j} \times \frac{{{{\sigma ^{\prime}}_i}}}{{\sqrt n }},j \in \{ 1,2, \ldots ,n\} ,i \in \{ 1,2, \ldots ,m\} ,$$

*b*_*i*_(*j*) cannot be determined unless *σ*_*i*_′ is provided in Eq. (3); hence, *σ*_*i*_′ is approximated by *σ*_*i*_, and the following expression is obtained^38^:

5 $${b^{\prime}_i}(j)={b_i}(j) - {s_j} \times \frac{{{\sigma _i}}}{{\sqrt n }},j \in \{ 1,2, \ldots ,n\} ,i \in \{ 1,2, \ldots ,m\} ,$$

The boundaries of the minority class were expanded based on the improved MCT algorithm using an RW strategy.

#### MCT-RW algorithm

**Fig. 1:**
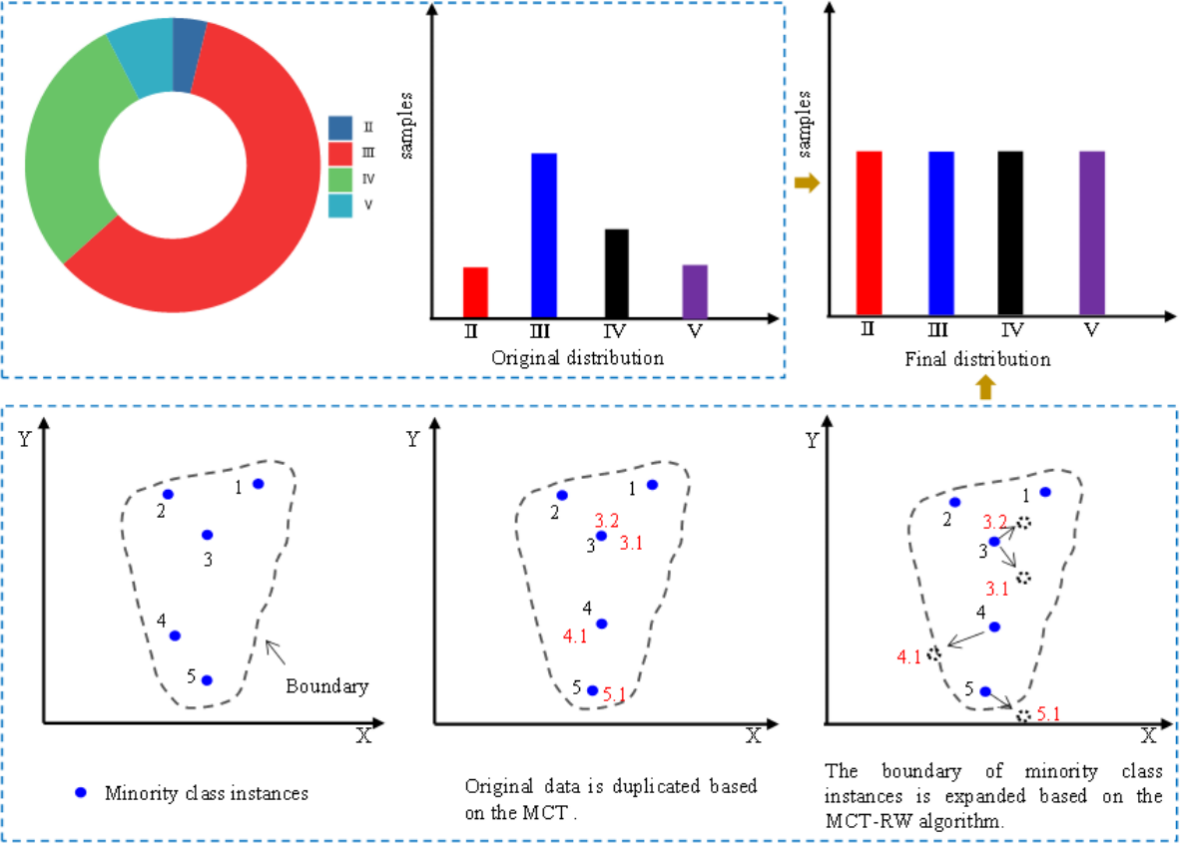
Schematic diagram of MCT and MCT-RW algorithm.

The training dataset is denoted as *P*, and the dataset representing the minority class is denoted as *P*_+_, comprising the elements $\left\{ {{x_1},…,{x_n}} \right\}.$ Each element *x*_*j*_ in *P*_+_ is characterized by *m* attributes, forming an *m*-dimensional vector within the *m*-dimensional attribute space. The set of attributes is labeled $B=\left\{ {{b_1},{b_2},…,{b_m}} \right\},$ and *b*_*i*_(*j*) symbolizes the value attributed to *b*_*i*_ for instance *x*_*j*_. The proposed MCT-RWO method is detailed in Algorithm 1, and a schematic is shown in Fig. 1.

**Algorithm 1:**
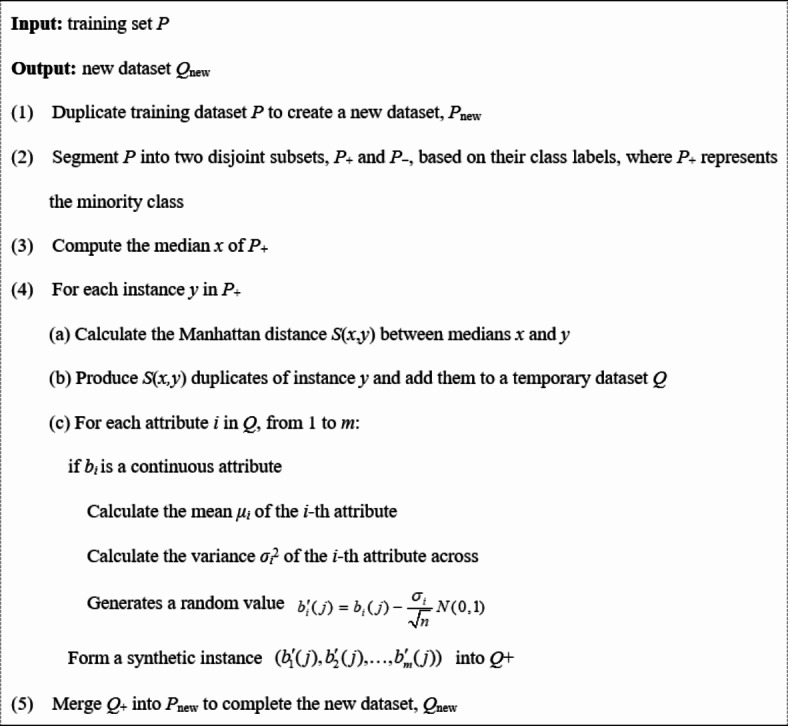
MCT-RW algorithm

### Basic and improved EWOAs

#### WOA

**Fig. 2:**
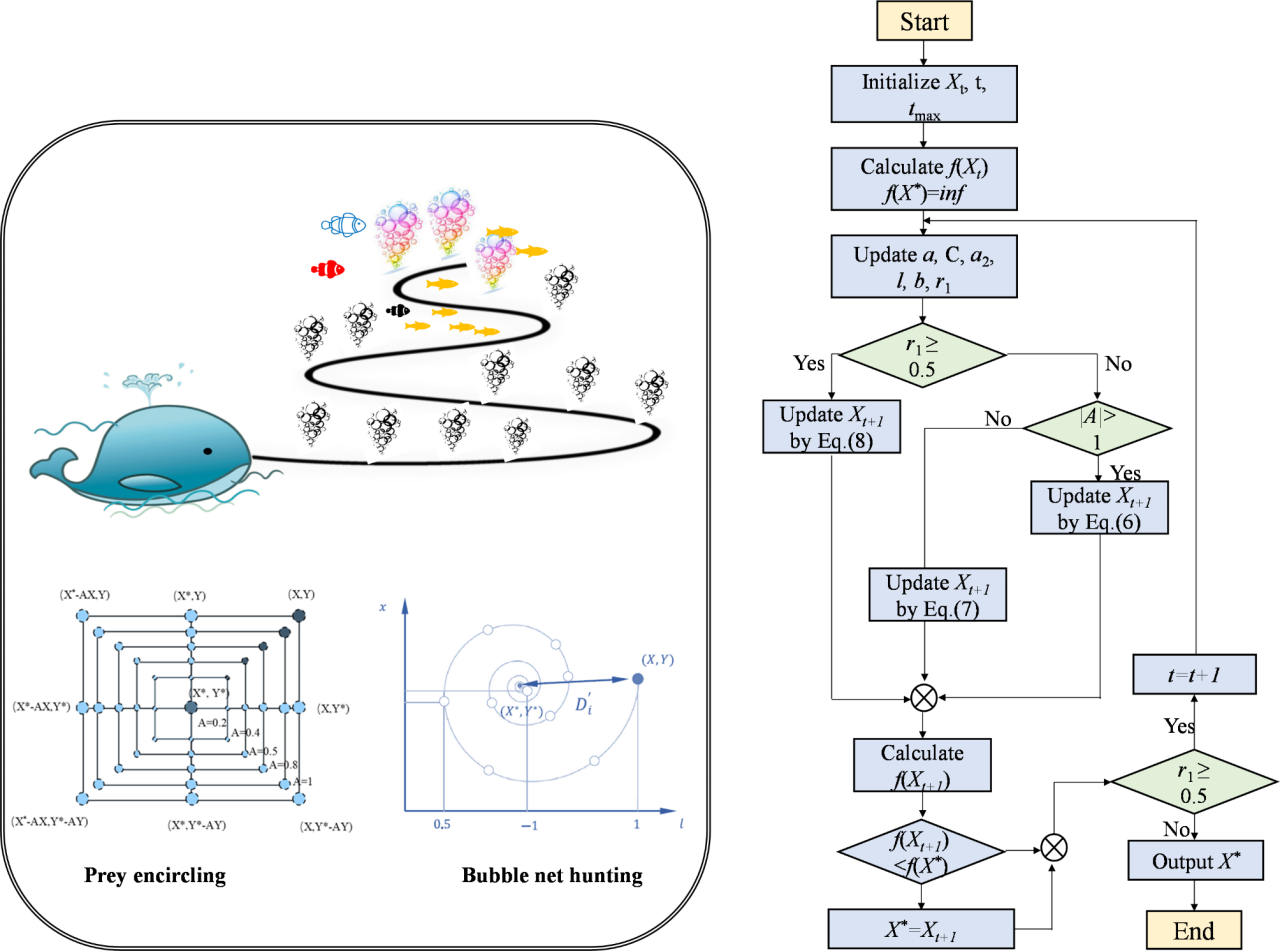
Standard WOA schematic and flowchart.

The WOA draws inspiration from the foraging behavior of humpback whales (as shown in Fig. 2). To model this behavior mathematically, Mirjalili and Lewis structured the WOA using three procedures^42^: (i) searching for prey (exploration stage) when |*A|* > 1, as formulated in Eq. (6); (ii) surrounding the prey when |*A|* < 1 (exploitation stage), as shown in Eq. (7); and (iii) creating a spiral bubble net to compensate for the prey exploitation stage, represented by Eq. (8).

If r_1_ < 0.5 and |A| > 1,

6 $${X_{t+1}}={X_{rand}} - A \times {D_{rand}},$$

where $${D_{rand}}=|C \times {X_{rand}} - {X_t}|.$$

If r_1_ < 0.5 and |A| ≤ 1,

7 $${X_{t+1}}={X^*} - A \times D,$$

where $$D=|C \times {X^*} - {X_t}|,$$$$A=2 \times a \times r - 2,$$$$a=2 - 2(t/t{}_{{\hbox{max} }}),$$ and $$C=2 \times r.$$

If r_1_ ≥ 0.5,

8 $${X_{t+1}}={X^*}+\cos (2\pi l) \times D^{\prime} \times {e^{bl}},$$

where $$l=({a_2} - 1) \times rand+1,$$$$D^{\prime}=|{X^*} - {X_t}|,$$ and $${a_2}=\frac{{ - 1 - t}}{{{t_{\hbox{max} }}}}.$$

Factor *A* oscillates randomly in the interval [–2, 2]. Concurrently, parameter *l* is a random variable within the interval [–1, 1]. Variable *t* signifies the ongoing iteration, with *t*_*max*_ denoting the cap on the iterations. The constant *b* is fine-tuned to the value of *l*. Lastly, *r* is a uniformly distributed random variable in the range [0, 1].

#### IEWOA

The EWOA [34] was proposed to enhance the simplicity and efficiency of the WOA. The EWOA introduces two main improvements: (1) employing a cosine function (with 1 > *cos* (2*πl*) > − 1) to modify the exploration and encircle stages, supplanting the previous factors where 2 > *A* > − 2, and (2) improving the exploitation stage by setting parameter *b* as a random integer from the range [0, 500].

If r_1_ < 0.5,

9 $${X_{t+1}}={X^*} - \cos (2\pi l) \times {D_{new}},$$

where $${D_{new}}=(C \times {X^*} - {X_t}).$$

If r_1_ ≥ 0.5,

10 $${X_{t+1}}={X^*}+\cos (2\pi l) \times D^{\prime} \times {e^{bl}},$$

where $$D^{\prime}=|{X^*} - {X_t}|.$$

The EWOA exhibits significant improvements over the original WOA; however, it still encounters certain challenges, such as the update strategy for parameter *l*. As a random variable, *l* facilitates the exploration of diverse regions within the search space by the algorithm during the exploration phase. It also indirectly determines the shape and behavior of the search trajectory during the exploitation stage. To endow the EWOA with a more versatile position updating mechanism, a novel calculation method for *l* was introduced, which is particularly reflected in parameter *a*_2_ as cited in reference^47^. The formula for this calculation is as follows:

11 $${a_2}=2 \times \left( {{e^{ - \left( {1+\frac{t}{{{t_{\hbox{max} }}}}} \right)}} - 1} \right).$$

This formula shows that the variable *a*_2_ undergoes an exponential decrement, transitioning from an initial value of − 1.26 to a terminal value of − 1.73, thereby delineating the bounds for parameter *l* to lie within the interval ranging from − 1.73 to 1.

**Fig. 3:**
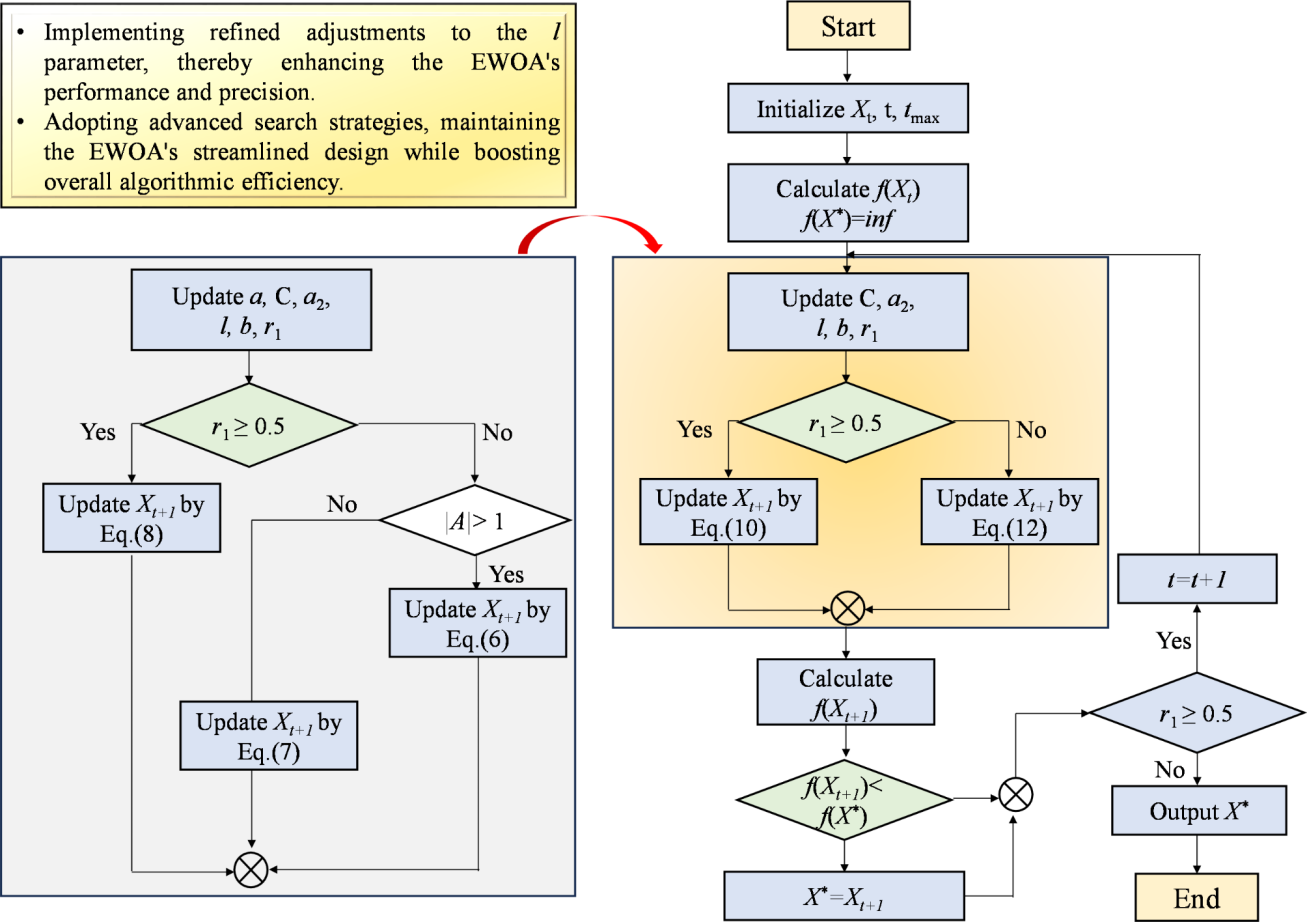
Flowchart of IEWOA.

To balance the exploration stage while navigating the search process, the algorithm now employs a sine function (where 1 > *sin*(2*πl*) > − 1) for encircling the target, as depicted in Eq. (12). This replaces the previous approach that used a cosine function (where 1 > *cos* (2*πl*) > − 1). The described adjustments to parameter *l* and the search process allowed the algorithm to maintain the streamlined design of the EWOA. At the same time, these adjustments elevated the overall efficacy of the algorithm. The conciseness of the IEWOA is exemplified by its reduced number of conditional statements and parameters, as shown in Fig. 3.

If r_1_ < 0.5,

12 $${X_{t+1}}={X^*} - \sin (2\pi l) \times {D_{new}}.$$

### LightGBM

**Fig. 4:**
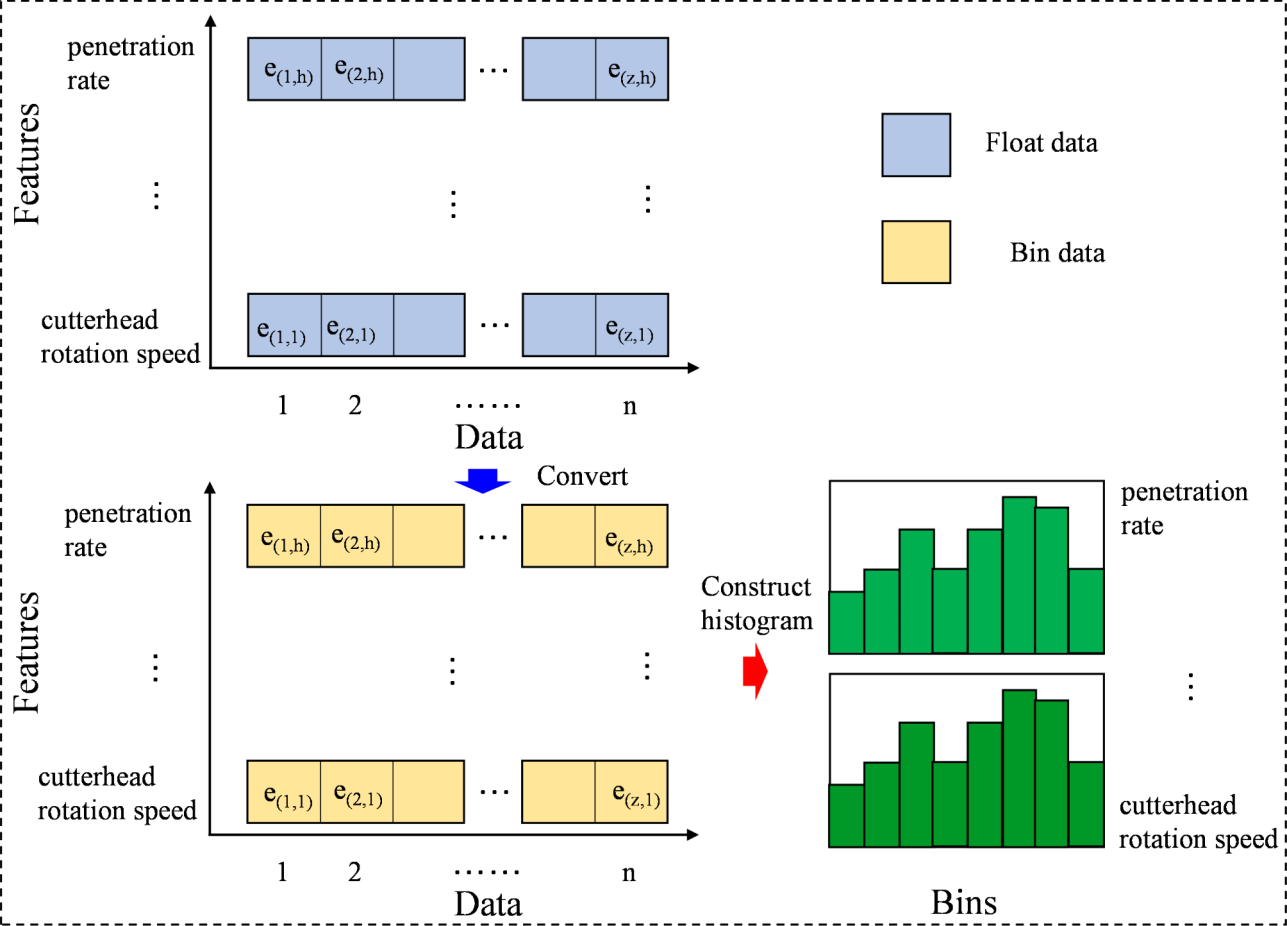
Schematic diagram of LightGBM model for rock mass class prediction.

LightGBM, an enhanced gradient boosting decision tree framework, addresses the inefficiency in training due to the high feature dimensions of traditional boosting algorithms^49,50^, as shown in Fig. 4. It accelerates training by using multithreaded parallel histograms. It also optimizes computational efficiency through gradient-based one-sided sampling and exclusive feature bundling. The former retains samples with large gradients while randomly selecting those with smaller gradients, and the latter reduces the features for histogram construction, thus lowering the complexity. Additionally, the leaf-wise growth strategy with depth limitation of LightGBM enhances the classification accuracy.

### Evaluation indicators

In this study, we employed the recall, precision, F_1_-score, and accuracy to assess the performance of various machine learning models. Specifically, the F_1_-score was calculated as the weighted average of precision and recall, as shown in Eqs. (13–16):

13 $$\text{R}\text{e}\text{c}\text{a}\text{l}\text{l}=\frac{{TP}}{{TP+FN}}$$

14 $$\Pr {\text{ecision=}}\frac{{TP}}{{TP+FP}}$$

15 $$F_{1} - {\text{score}} = \frac{{2 \times {\text{Precision}} \times {\text{Recall}}}}{{{\text{Precision}} + {\text{Recall}}}}$$

16 $$\text{Accuracy} = \frac{{TP + TN}}{{TP + FN + FP + TN}}.$$

These equations define TP, FP, TN, and FN as the numbers of true positives, false positives, true negatives, and false negatives, respectively.

## Framework of the IEWOA-MCT-RW-LightGBM forecast model

**Fig. 5:**
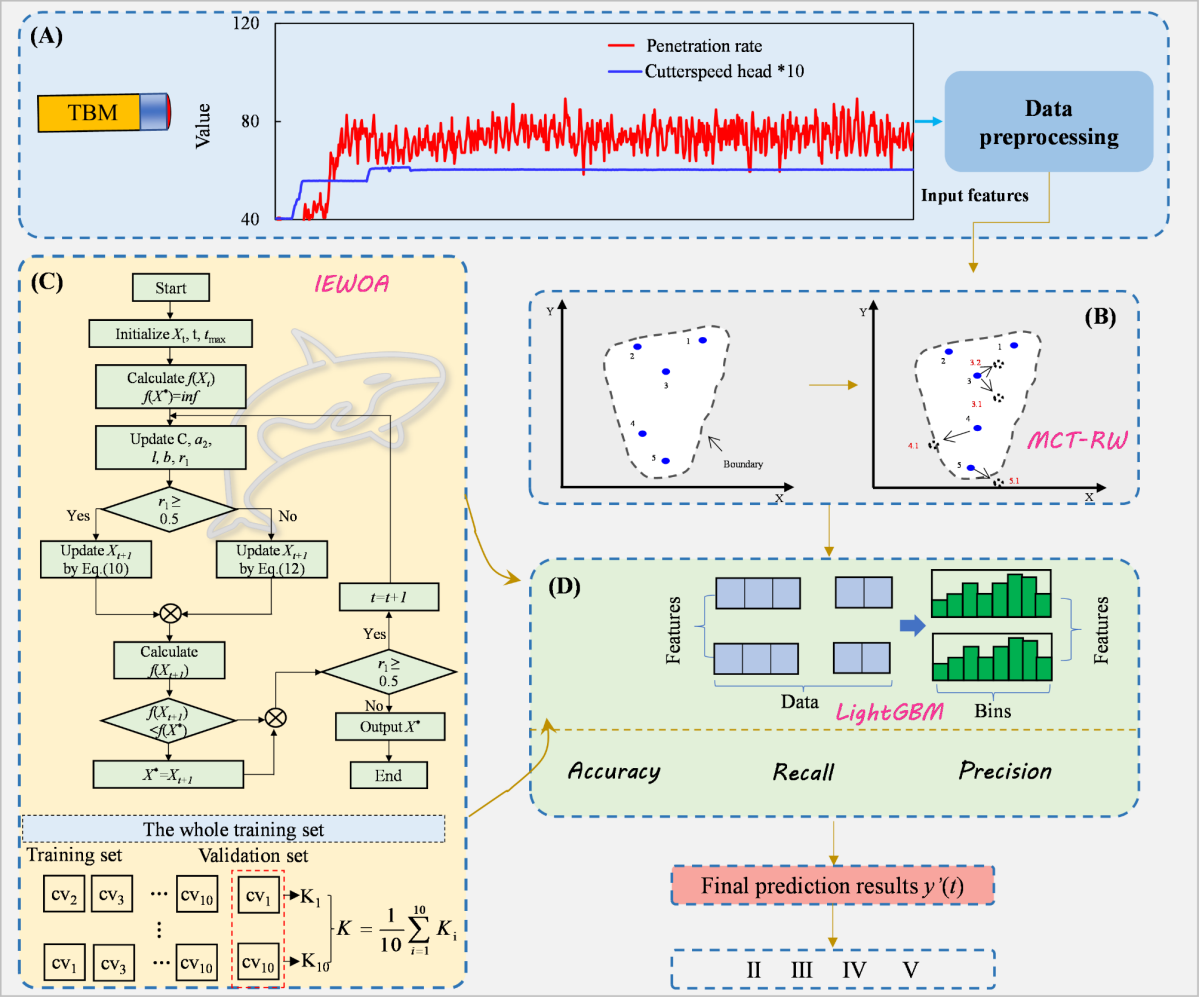
Workflow of the rock mass class prediction process.

The flowchart of the rock mass class prediction process is depicted in Fig. 5 and consists of four modules: (A) a TBM data preprocessing module, (B) the proposed MCT-RW data balancing module, (C) an IEWOA optimization model module, and (D) a model construction and evaluation module. The detailed explanations of each module are as follows.

(A) TBM data preprocessing module: This module extracts effective excavation cycles from the TBM construction data, selects 12 input features, and uses the means of the effective data from the TBM excavation cycles as inputs.
(B) Proposed MCT-RW data-balancing module: Considering that the MCT can only replicate sample data without expanding the boundaries of the minority class samples, the MCT-RW algorithm is utilized.
(C) IEWOA optimization model module: To address the shortcomings of the whale optimization algorithm, this module employs a modified parameter *l* and different search strategies, facilitating the avoidance of local optima and securing optimal hyperparameters.
(D) Model construction and evaluation module: The applicability of different models and data-balancing algorithms for predicting the rock mass class was evaluated using four metrics: accuracy, recall, precision, and F_1_-score.

## Case study data

### Project overview

**Fig. 6:**
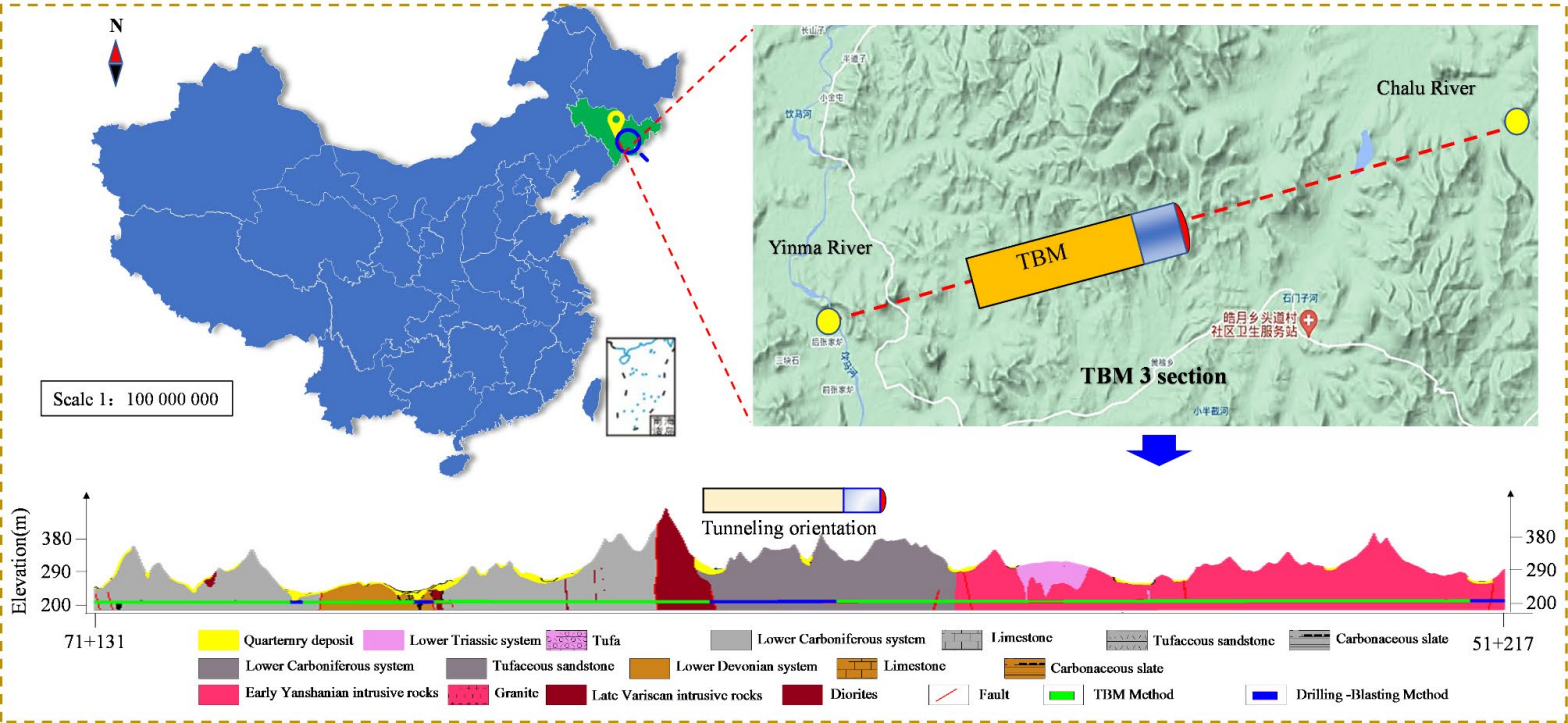
Planimetric map of the Yinsong water diversion project.

The Yinsong water diversion Project of Jinlin Province is featured in the 13th National Five-Year Plan (2016–2020) of China. It is a key water conservation initiative among the 172 major projects. This study was focused on section TBM-3 of the project, which spans 263 km in length (see Fig. 6), extending from Yinma River to Chalu River, with a TBM boring length of 17.5 km. The tunnel, with a 7.93 m diameter and depth ranging from 85 m to 260 m, traverses the terrain of hills and valleys with steeply inclined rocks. This geologically diverse and variable area poses significant challenges in the design of TBM tunnels.

### Rock mass classification method

In this study, the rock mass class was assessed using the Chinese hydropower classification method. The Chinese hydropower classification method considers rock strength, rock mass integrity, and discontinuity conditions and assigns positive values^20^. The groundwater conditions and attitude of the main discontinuity plane serve as corrective factors, marked with negative values. The sum of these five factors is the fundamental criterion for classification.

Class I represents extremely hard, intact rock masses, whereas Class V consists of extremely weak and mostly broken rocks. To examine the correlation between the rock mass classes and TBM tunneling parameters, a dataset was compiled. It comprised 285 samples (4%) from Class II, 4465 samples (59%) from Class III, 2191 samples (29%) from Class IV, and 564 samples (8%) from Class V. Notably, Class I rock masses were absent in this project.

Intact rock strength (*A*), rock mass intactness degree (*B*), and discontinuity conditions (*C*) are identified as primary influence factors in the HC method, with each factor assigned a positive value. Additionally, ground water condition (*D*) and the orientation of the main discontinuity plane (*E*) serve as correction factors, assigned negative values. The cumulative score *T = A + B + C + D + E*, a composite index, is derived from these five factors using an accumulation approach. Rock mass classification is then comprehensively determined by incorporating the strength-stress ratio S, which accounts for the impact of stress conditions on the stability of the surrounding rock. The strength-stress ratio S is computed using Eq. (17). Table 1 illustrates the rock mass classification based on the HC method.

17 $$S=\frac{{{R_c} \times {K_v}}}{{{\sigma _m}}}$$

Where *S* represents the strength-stress ratio, *R*_*c*_ is the uniaxial compressive strength of intact saturated rock (MPa), *K*_*v*_ denotes the intactness index of the rock mass, and *σ*_*m*_ is the maximum principal stress of the surrounding rock (MPa).

**Table 1:** Classification of rock mass based on the HC method.

Class	I	II	III	IV	V
*T*	> 85	85 ≥ *T* > 65	65 ≥ *T* > 45	45 ≥ *T* > 25	≤ 25
*S*	> 4	> 4	> 2	> 2	-

### Input features

**Fig. 7:**
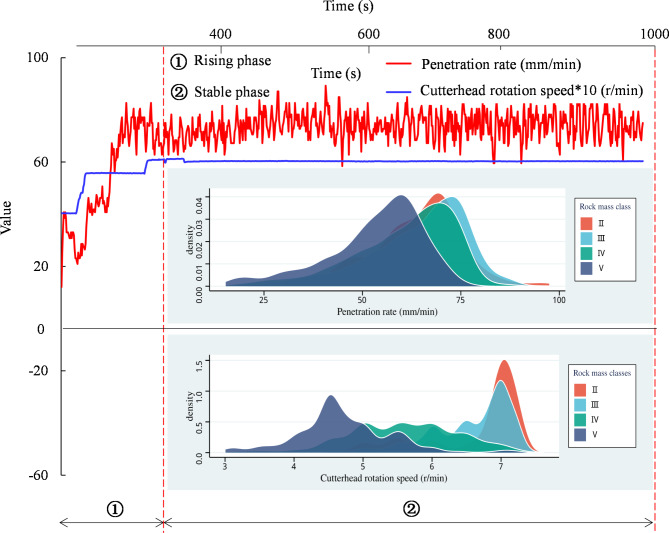
Schematic diagram of the rising and stable stages of the TBM tunneling cycle.

TBM tunneling data are usually recorded in continuous cycles, with 1.8 m per tunneling cycle being ideal. However, this varies greatly owing to adverse geological conditions. To ensure accuracy, 7505 cycles with tunneling footage exceeding 0.05 m were gathered, excluding shorter drills that could have led to data inaccuracies. Each tunneling cycle primarily consisted of four phases: starting, rising, stable, and shutdown. The algorithm for dividing the tunneling cycle into four stages is detailed in^20^. The starting and shutdown stages were considered invalid. The rising and stable stages are illustrated in Fig. 7. Owing to the large number of TBM tunneling cycles and the extensive data involved, the means of the rising and stable stages were selected as the inputs. Clear differences in the density distribution of the tunneling parameters under different rock mass classes can be observed in Fig. 7. Next, the selected input features are introduced.

**Fig. 8:**
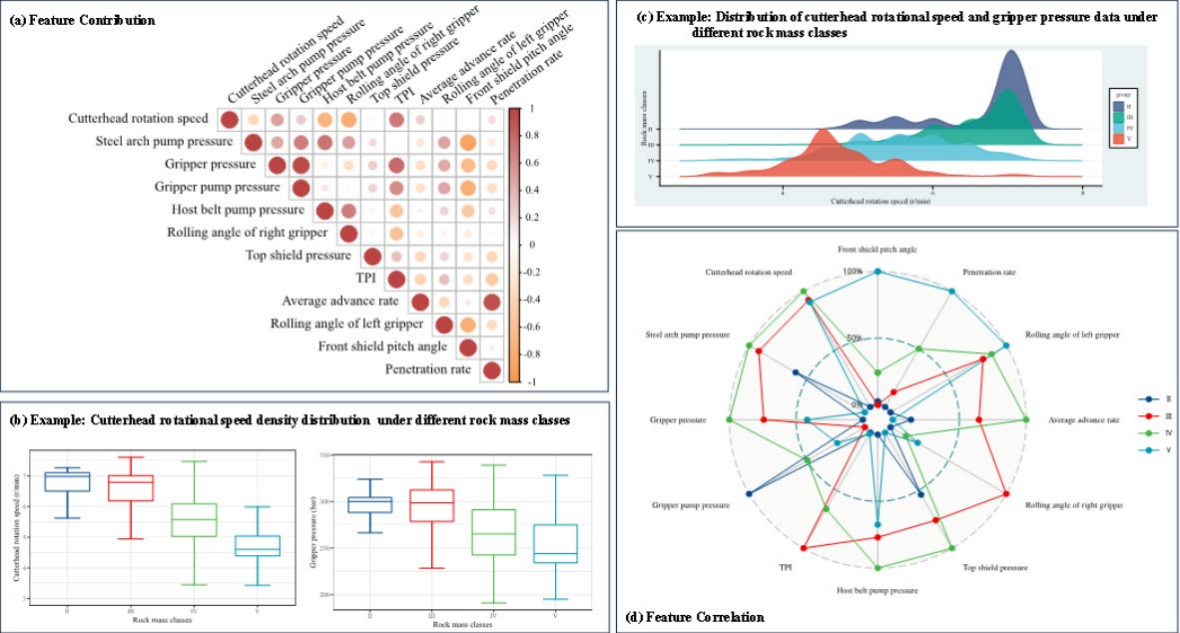
Input feature correlation, data distribution and feature contribution.

In a previous study, 12 key features were identified from a set of 21 for predicting rock mass classes^20^ by leveraging LightGBM and SHapley Additive exPlanations^51^. These features included the cutterhead rotation speed, steel arch pump pressure, gripper pressure, gripper pump pressure, host belt pump pressure, rolling angle of the right gripper, top shield pressure, torque penetration index (TPI), average advance rate, rolling angle of the left gripper, front shield pitch angle, and penetration rate. To clearly demonstrate the data characteristics and distribution of the input features, we compiled data on feature correlations and boxplot distributions. We used examples such as the cutterhead rotation speed and ripper pressure, and ridge plots of typical features, like the cutterhead rotation speed, are also provided. The contributions of these features under different rock mass classes are shown in Fig. 8. Specific information on histograms of other input features and the linear relationships between different input features, represented in the form of heatmaps, can be found in the relevant literature^20^. Statistical details of the input features are provided in Table 2.

**Table 2:** Statistics of input features in predicting rock mass classes.

Input features	Mean	Standard deviation	Minimum	Maximum
Cutterhead rotation speed (r/min)	6.14	0.90	3.00	7.61
Steel arch pump pressure (bar)	194.39	2.80	138.07	199.22
Gripper pressure (bar)	283.79	31.55	191.00	342.79
Gripper pump pressure (bar)	299.10	25.71	225.09	351.46
Host belt pump pressure (bar)	109.87	5.75	90.42	159.06
Rolling angle of the right gripper (°)	1.06	1.03	-5.00	4.38
Top shield pressure (bar)	48.05	13.63	0.86	91.60
TPI (kN·mm^−1^)	4.10	1.69	0.77	19.06
Average advance rate (m/h)	3.16	1.07	0.03	13.67
Rolling angle of the left gripper (°)	-0.62	1.03	-4.34	3.62
Front shield pitch angle (°)	0.53	0.17	-0.08	1.08
Penetration rate (mm/min)	62.51	13.05	15.28	97.43

## Model establishment and validation

### Validation of the IEWOA performance

The effectiveness of the newly developed IEWOA was assessed using the 15 benchmark functions listed in Table 3. Comparisons were made between the IEWOA results and those of other algorithms, including the GWO^40^, LWOA^45^, EWOA^46^, and standard WOA^42^.

Table 4 lists the means and Stds of the evaluated benchmark functions. Each benchmark function was subjected to an optimization algorithm with 500 iterations. Throughout this procedure, the algorithm used 30 particles. Subsequently, the mean, Std, and best values were calculated based on the results of the 30 independent trials. The data in Table 4 demonstrate that the IEWOA achieves superior performance compared with the other algorithms. This advantage is particularly pronounced when applied to benchmark functions F1, F3, F4, F6, F10, F12, F13, and F14, demonstrating the significant strengths of the IEWOA in these scenarios. On the benchmark function F2, the LWOA exhibits the most favorable mean and Std; however, the IEWOA possesses the best value. On benchmark function F8, the GWO algorithm shows superiority in terms of the Std and best value, whereas the mean achieved by the IEWOA is superior. On benchmark function F9, the GWO algorithm surpasses the others. Compared to the EWOA, the IEWOA demonstrates equal or superior performance on 15 × 15 benchmark functions.

**Table 3:** GWO algorithm, WOA, LWOA, and EWOA results for 15 benchmark functions.

Function	Range
$${F_1}(x)=\sum\nolimits_{{i=1}}^{D} {|{x_i}} |+\prod\nolimits_{{i=1}}^{D} {|{x_i}|}$$	[–10, 10]
$${F_2}(x)=\sum\nolimits_{{i=1}}^{D} {\left[ {100{{\left( {{x_{i+1}} - {x_i}^{2}} \right)}^2}+{{\left( {{x_i} - 1} \right)}^2}} \right]}$$	[–30, 30]
$${F_3}(x)=\mathop {\hbox{max} }\limits_{{1 \leqslant i \leqslant D}} |{x_i}|$$	[–100, 100]
$${F_4}(x)=\sum\nolimits_{{i=1}}^{D} {ix_{i}^{4}} +random[0,1)$$	[–1.28, 1.28]
$${F_5}(x)={\left( {\left\lfloor {|{x_i}+0.5|} \right\rfloor } \right)^2}$$	[–100, 100]
${F_6}(x)={\left( {\frac{1}{{500}}+\sum\nolimits_{{j=1}}^{{25}} {\frac{1}{{j+\sum\nolimits_{{i=1}}^{2} {{{({x_i} - {a_{ij}})}^6}} }}} } \right)^{ - 1}}$	[–65.536, 65.536]
$${F_7}(x)={\left( {{x_2} - \frac{{5.1x_{1}^{2}}}{{4{\pi ^2}}}+\frac{{5{x_1}}}{\pi } - 6} \right)^2}+10\left( {1 - \frac{1}{{8\pi }}} \right)\cos ({x_1})+10$$	[–5, 10]
$${F_8}(x)=4x_{1}^{2} - 2.1x_{1}^{4}+\frac{1}{3}x_{1}^{6}+{x_1}{x_2} - 4x_{2}^{2}+4x_{2}^{4}$$	[–5, 5]
$${F_9}(x)= - \sum\limits_{{i=1}}^{m} {{c_i}\exp \left( { - \sum\limits_{{j=1}}^{n} {{a_{ij}}{{\left( {{x_j} - {p_{ij}}} \right)}^2}} } \right)}$$	[0, 1]
$${F_{10}}(x)=\sum\nolimits_{{i=1}}^{D} {|{x_i}\sin ({x_i})} +0.1{x_i}|$$	[–10, 10]
$$F_{{11}} (x) = \sum\limits_{{i = 1}}^{D} {\sin (x_{i} )} \sin ^{{2m}} \left( {\frac{{ix_{i}^{2} }}{\pi }} \right)$$	[0, ]
$${F_{12}}(x)=\exp \left( { - 0.5\sum\limits_{{i=1}}^{D} {x_{i}^{2}} } \right)$$	[–1, 1]
$${F_{13}}(x)=0.5+\frac{{{{\sin }^2}\left( {\sqrt {x_{1}^{2}+x_{2}^{2}} - 0.5} \right)}}{{{{[1+0.001(x_{1}^{2}+x_{2}^{2})]}^2}}}$$	[–100, 100]
$${F_{14}}(x)= - \frac{{1+\cos \left( {12\sqrt {x_{1}^{2}+x_{2}^{2}} } \right)}}{{0.5\left( {x_{1}^{2}+x_{2}^{2}} \right)+2}}$$	[–5.12, 5.12]
$${F_{15}}(x)=\frac{\pi }{D}\left[ {10{{\sin }^2}{{(\pi {y_1} - 1)}^2}} \right]\left[ {1+10{{\sin }^2}(\pi {y_{1+1}})+{{(yD - 1)}^2}} \right]$$	[–10, 10]

**Table 4:** Statistical results (mean, Std, and best value) for 15 benchmark functions.

F	-	GWO	WOA	LWOA	EWOA	IEWOA
F1	Mean	2.90E–16	5.49E–54	4.04E–02	3.59E–197	**1.17E**–**204**
	Std	1.59E–16	2.86E–53	2.21E–02	0.00E + 00	**0.00E + 00**
	Best	3.60E–17	6.39E-64	8.98E–03	4.04E–222	**4.10E**–**235**
F2	Mean	2.70E + 01	1.19E + 01	**1.96E**–**02**	1.35E–01	1.58E–01
	Std	6.60E-01	1.30E + 01	**1.74E**–**02**	2.46E–01	3.65E–01
	Best	2.61E + 01	2.83E–02	1.35E–04	2.46E–04	**1.56E**–**05**
F3	Mean	7.70E–07	1.83E–09	2.18E–03	4.12E–203	**6.92E**–**208**
	Std	8.00E–07	9.55E–09	1.96E–03	0.00E + 00	**0.00E + 00**
	Best	7.14E–08	1.68E–17	3.52E–04	2.60E–231	**1.57E**–**237**
F4	Mean	2.12E–03	6.77E–04	5.20E–03	1.72E–04	**1.66E**–**04**
	Std	1.43E–03	1.09E–03	5.36E–03	2.26E–04	**1.35E**–**04**
	Best	7.58E–04	9.65E-06	7.09E–04	1.18E–05	**6.16E**–**06**
F5	Mean	**0.00E + 00**	**0.00E + 00**	**0.00E + 00**	**0.00E + 00**	**0.00E + 00**
	Std	**0.00E + 00**	**0.00E + 00**	**0.00E + 00**	**0.00E + 00**	**0.00E + 00**
	Best	**0.00E + 00**	**0.00E + 00**	**0.00E + 00**	**0.00E + 00**	**0.00E + 00**
F6	Mean	4.23E + 00	3.12E + 00	2.73E + 00	2.38E + 00	**1.69E + 00**
	Std	4.02E + 00	3.56E + 00	3.02E + 00	2.26E + 00	**1.42E + 00**
	Best	**9.98E**–**01**	**9.98E**–**01**	**9.98E**–**01**	**9.98E**–**01**	**9.98E**–**01**
F7	Mean	3.98E–01	3.98E–01	3.98E–01	3.98E–01	**3.98E**–**01**
	Std	**2.05E**–**06**	1.18E–04	1.07E–05	3.45E–04	8.02E–04
	Best	**3.98E**–**01**	**3.98E**–**01**	**3.98E**–**01**	**3.98E**–**01**	**3.98E**–**01**
F8	Mean	–1.03E + 00	–1.03E + 00	–1.03E + 00	–1.03E + 00	–**1.03E + 00**
	Std	3.43E–08	**8.49E**–**10**	5.25E–06	3.98E–06	1.73E–06
	Best	–**1.03E + 00**	–**1.03E + 00**	–**1.03E + 00**	–**1.03E + 00**	–**1.03E + 00**
F9	Mean	–**3.28E + 00**	–3.08E + 00	–3.17E + 00	–2.86E + 00	–2.85E + 00
	Std	**6.47E**–**02**	1.91E–01	1.03E–01	3.00E–01	3.19E–01
	Best	–**3.32E + 00**	–**3.30E + 00**	–**3.32E + 00**	–**3.29E + 00**	–**3.21E + 00**
F10	Mean	3.89E–04	6.49E–56	1.17E–01	1.10E–197	**1.33E**–**199**
	Std	4.91E–04	2.37E–55	6.08E–01	0.00E + 00	**0.00E + 00**
	Best	3.81E–17	3.22E–64	3.66E–04	2.23E–222	**2.07E**–**233**
F11	Mean	–1.80E + 00	–1.77E + 00	–1.80E + 00	–1.64E + 00	–1.68E + 00
	Std	4.68E-06	1.44E–01	3.60E–05	2.74E–01	2.52E–01
	Best	–1.80E + 00	–1.80E + 00	–1.80E + 00	–1.80E + 00	–1.80E + 00
F12	Mean	–1.00E + 00	–1.00E + 00	–1.00E + 00	–1.00E + 00	–1.00E + 00
	Std	2.03E–17	0.00E + 00	2.32E–05	0.00E + 00	0.00E + 00
	Best	–1.00E + 00	–1.00E + 00	–1.00E + 00	–1.00E + 00	–1.00E + 00
F13	Mean	2.46E–03	4.36E–03	2.46E–03	2.46E–03	2.46E-03
	Std	3.23E–10	5.70E–03	4.49E–09	3.43E–12	8.44E-16
	Best	2.46E–03	2.46E–03	2.46E-03	2.46E–03	2.46E–03
F14	Mean	–9.94E–01	–9.85E–01	–9.87E–01	–1.00E + 00	–1.00E + 00
	Std	1.91E–02	2.70E–02	2.55E–02	0.00E + 00	0.00E + 00
	Best	–1.00E + 00	–1.00E + 00	–1.00E + 00	–1.00E + 00	–1.00E + 00
F15	Mean	4.02E–02	1.09E–03	6.81E–06	1.88E–04	1.40E–04
	Std	2.07E–02	7.94E–04	8.59E–06	3.79E–04	2.91E–04
	Best	1.10E–02	1.96E–05	1.85E–07	5.15E–07	1.24E–06

The bold numbers are the optimal results of the compared optimization algorithms.

**Fig. 9:**
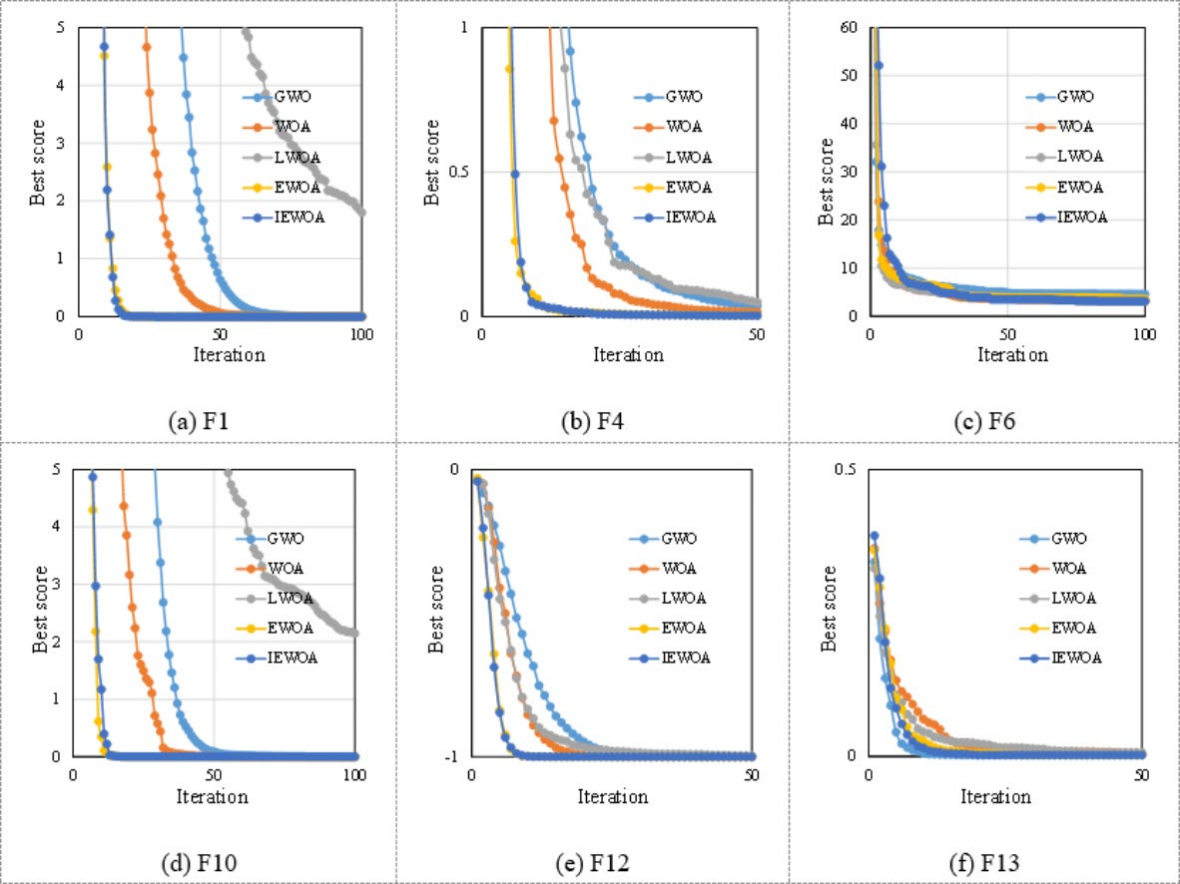
Convergence behavior of tested GWO algorithm, WOA, LWOA, EWOA, and IEWOA.

To highlight the significant differences between the proposed IEWOA and the other algorithms, Fig. 9 illustrates the iterative process on benchmark functions F1, F3, F4, F6, F10, F12, F13, and F14. Iteration processes of either 50 or 100 steps were selected for clearer observation and comparison of the convergence characteristics of the different algorithms. In most cases, the IEWOA demonstrates a faster convergence rate than the other algorithms. Although the IEWOA displays a convergence curve comparable to that of the EWOA, it attains notable performance improvements by optimizing parameter *l* and refining its search strategies. Considering the challenge of numerous hyperparameters in LightGBM, the next step involves utilizing algorithms such as the IEWOA for model optimization.

### Predictive results of the proposed hybrid algorithm

Previous research demonstrated the effectiveness of LightGBM in predicting rock mass classifications, particularly its strengths in managing high-dimensional data and addressing class imbalance^20^. To further enhance its performance, we used the WOA and its improved version, IEWOA. These algorithms were applied to fine-tune key hyperparameters, including maximum depth, number of estimators, number of leaves, and learning rate. The maximum depth parameter is optimized within the range of (1, 50] and controls the depth of decision trees. This allows the model to capture intricate data patterns while minimizing the risk of overfitting. The number of estimators is set between (1, 500] and determines the total number of trees in the ensemble. While more trees generally improve predictive accuracy, this range ensures a balance between performance gains and computational efficiency. The number of leaves, optimized from (1, 200], dictates the detail level of data splits, which is essential for identifying subtle patterns without compromising model stability. The learning rate, set within [0.001, 0.5], manages how quickly the model adapts during training. It provides a trade-off between convergence speed and prediction accuracy, refining model performance effectively. Table 5 outlines the specific ranges for these parameters, demonstrating the deliberate balance needed to optimize LightGBM for complex tunneling scenarios.

A 5-fold cross-validation approach was employed during optimization to minimize overfitting risks and prevent convergence to suboptimal solutions. This method ensures that the selected hyperparameters improve the model’s current performance. It also enhances the model’s ability to generalize to new and varied datasets. By strategically optimizing these parameters, the model is better positioned to accurately classify rock masses, thus improving its effectiveness and applicability in real-world tunneling projects.

**Table 5:** Hyperparameters of LightGBM optimization for rock mass class prediction.

Hyperparameter	Explanation	Value	Type
Maximum depth	Maximum tree depth for base learners	(1, 50]	Integer
Number of estimators	Number of boosted trees to fit	(1, 500]	Integer
Number of leaves	Maximum tree leaves for base learner	(1, 200]	Integer
Learning rate	Boosting learning rate	[0.001, 0.5]	Continuous

This section compares the predictive performances of the optimized models applied to the original and balanced datasets. Conventionally, a dataset is divided into training and testing subsets in a ratio of 80:20. To mitigate the potential predictive biases engendered by the stochastic dispersal of data, the dataset was subjected to 10 equal-probability random allocations in this study. Subsequently, the average was computed from the model predictions derived from these allocations. Repeated random allocation and averaging of the dataset are essential for overcoming the inherent uncertainties associated with the outcomes derived from single random partitioning.

Data balancing focuses on ensuring equal sample sizes across different classes. Specifically, it aims to adjust the sample counts of minority classes, such as Classes II, IV, and V, to match those of the more common Class III. This approach is crucial for balancing the dataset and preventing any tendency of the model to favor a class with a larger number of samples, thus ensuring a more balanced and fair analysis.

**Fig. 10:**
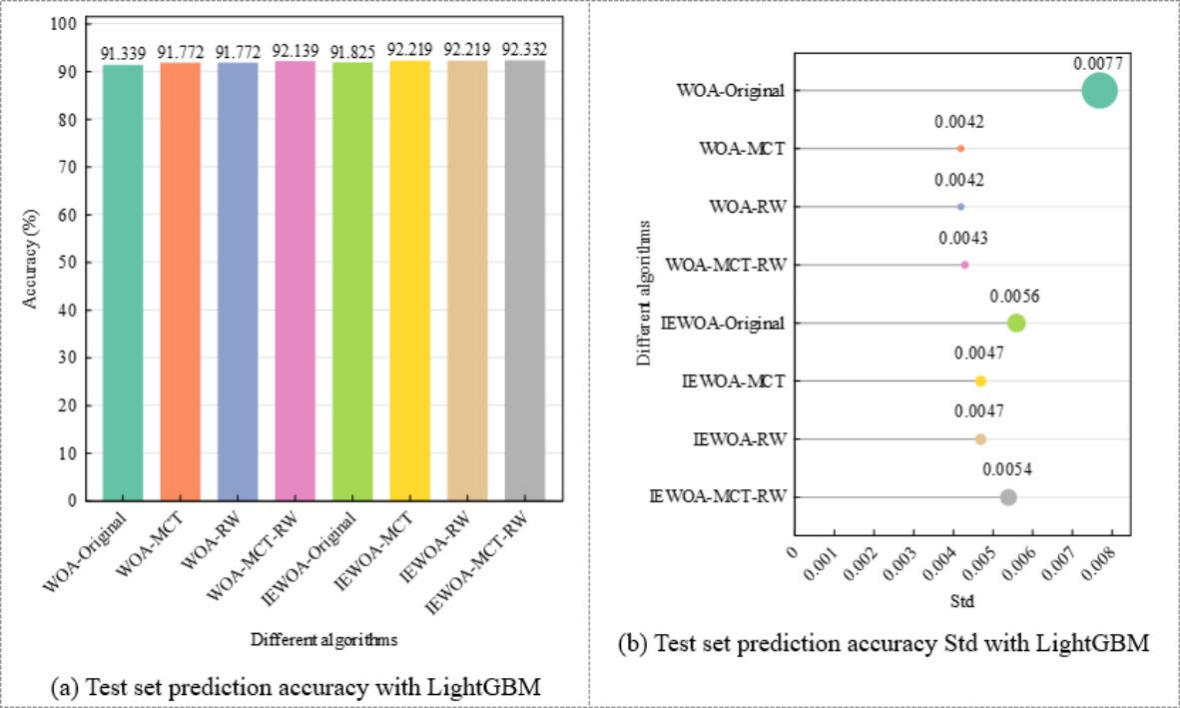
Comparative analysis of LightGBM predictions with various optimization and data balancing algorithms.

**Fig. 11:**
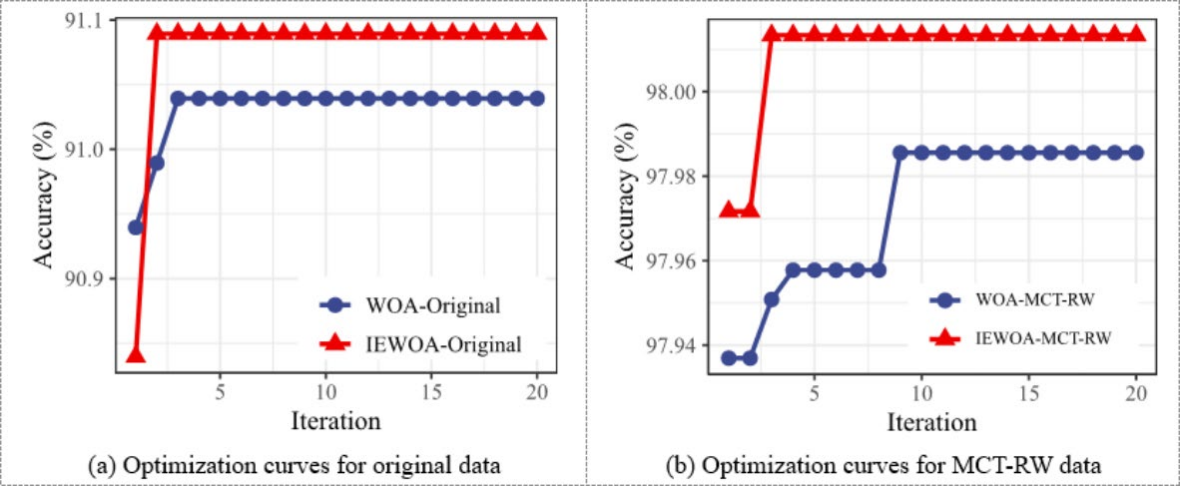
Iteration curves of WOA and IEWOA optimizations.

As shown in Fig. 10a,b, the prediction results obtained using the IEWOA-MCT-RW algorithm are optimal (92.332%), although its Std is slightly higher than those of IEWOA-MCT and IEWOA-RW, and the predictions based on the IEWOA significantly outperform those based on the WOA. In “MCT” section, the MCT algorithm uses the Manhattan distance instead of the Euclidean distance. Additionally, a comparison of these two distance metrics shows that the predictive results using the Manhattan distance outperform those using the Euclidean distance by an average of 0.31%. Figure 11a,b demonstrate that for the same dataset, the optimization results of the IEWOA are superior to those of the WOA. Analysis of the optimization process indicated that the model tended to stabilize within approximately 20 steps.

Despite the relatively minor differences in performance among the various models, enhancing the model performance plays a pivotal role in the TBM construction process. The application of automated real-time predictive analytics for the rapid development of targeted strategies is crucial for ensuring the efficiency and safety of TBM tunneling operations.

**Fig. 12:**
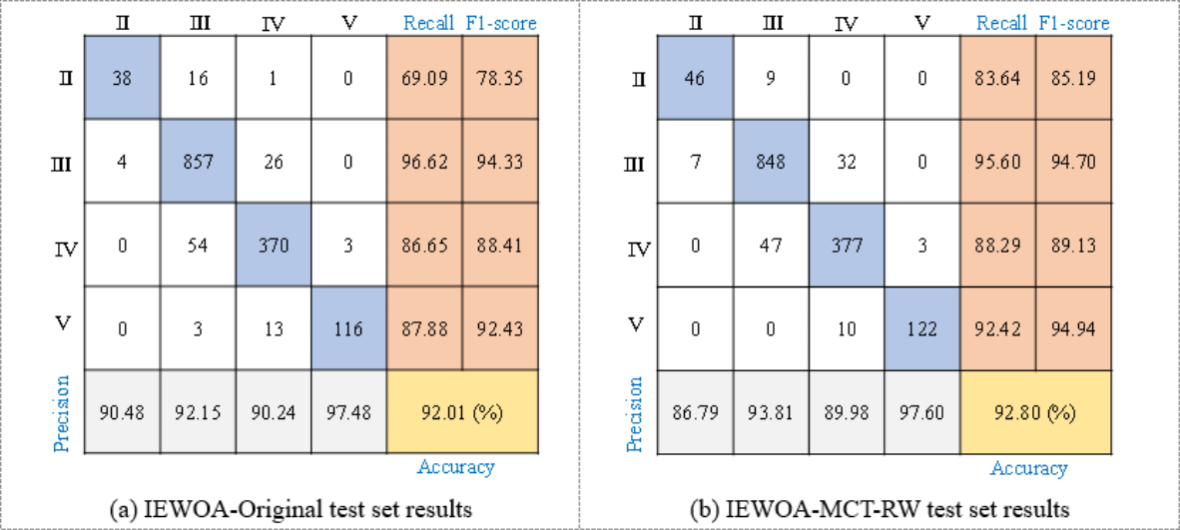
Confusion matrix of test set prediction of LightGBM trained by original data and MCT-RW balanced data.

Additionally, a significant impediment to the enhancement of the model performance is the notable similarity between Classes II and III, as well as that between Classes IV and V within the input features. In this context, the possibility of merging Class II with Class III and Class IV with Class V is imperative. Assessing how such consolidation might affect the predictive results is equally vital. A comprehensive analysis and in-depth discussion of these potential impacts are presented in Sect. 5.3. To illustrate the variability of the test results, two models were evaluated using the same test set. One model was trained with the original dataset and achieved an accuracy of 92.01%. The other model was trained with a balanced dataset and achieved an accuracy of 92.80%. This outcome, representing only one of the random trials, highlights the inherent variability in model testing. As depicted in Fig. 12, data balancing significantly boosts the recall and F_1_-score for Classes II, IV, and V, yet concurrently exhibits a discernible 1% decrease in recall for Class III. This drop is primarily ascribed to the implementation of the MCT-RW algorithm, which incorporates a RW strategy, thereby leading to the classification of Class III data as Class II or IV data. These observations highlight the intricate dynamics among data balancing, algorithmic selection, and inherent data similarities, which are pivotal in refining the efficacy of predictive models.

### Validation of the applicability of the proposed algorithm in a new tunnel

Accurate prediction of rock mass classes is crucial for strategically planning TBM construction schedules, as it directly affects the selection of tunneling parameters. When actual construction data is unavailable before a project begins, it is essential to rely on methodologies or models from similar TBM projects. This is especially true for projects with comparable features, such as cutterhead diameter. This section examines the effectiveness of the LightGBM model in enhancing the precision of rock mass class predictions. The model was trained on both original and balanced data from the Yinsong project for future tunnel construction under similar TBM configurations.

The construction of validation tunnels typically employs an open-type TBM featuring a cutter with a diameter of 7.8 m. For the validation process, data collection covered a section constructed by the TBM, extending over a length of 755 m. This included 343 tunneling cycles, with 2 samples from Class II, 293 from Class III, and 48 from Class IV. Regarding the input features for predicting the rock mass classes within the validation tunnel, seven crucial tunneling parameters were obtained. These included the cutter head rotation speed, gripper pressure, gripper pump pressure, TPI, host belt pump pressure, top shield pressure, and average penetration rate.

**Fig. 13:**
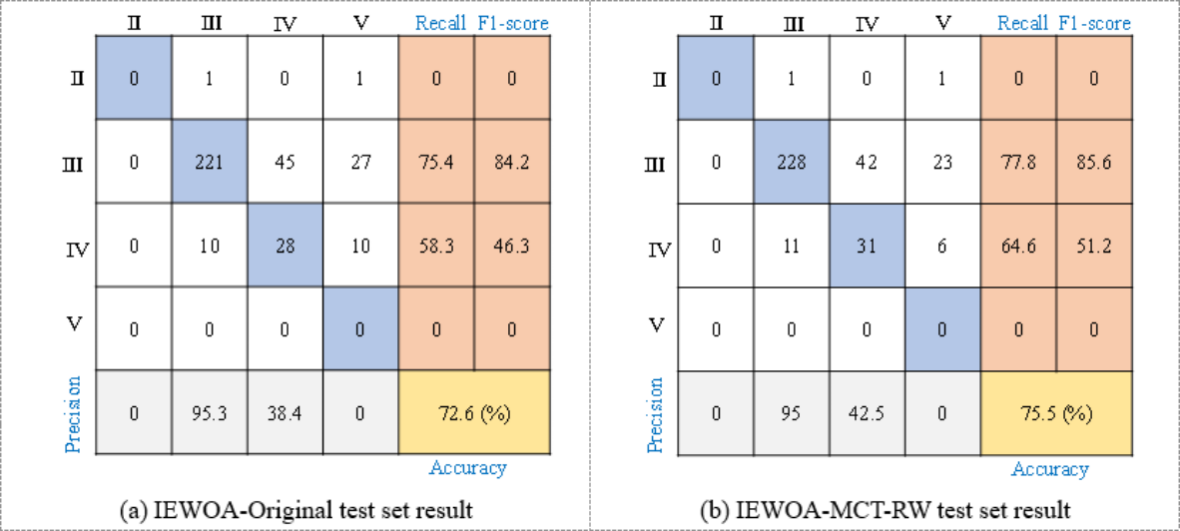
Confusion matrix of test set prediction of LightGBM for new TBM project.

Using the balanced dataset from the Yinsong project to train LightGBM significantly improved the model’s performance when predicting rock mass classes in new tunnel construction projects, achieving an accuracy of 75.5% compared to 72.6% when trained on the original dataset, as shown in Fig. 13. This improvement is especially evident in predicting Class III and Class IV rock masses, where the model showed notable gains in recall and F1-score. The data balancing approach allowed the model to better capture the characteristics of minority classes, enhancing its predictive reliability in new project scenarios. However, both models still struggled to accurately identify Class II rock masses due to the limited sample size, highlighting the need for further refinements in handling underrepresented classes.

**Fig. 14:**
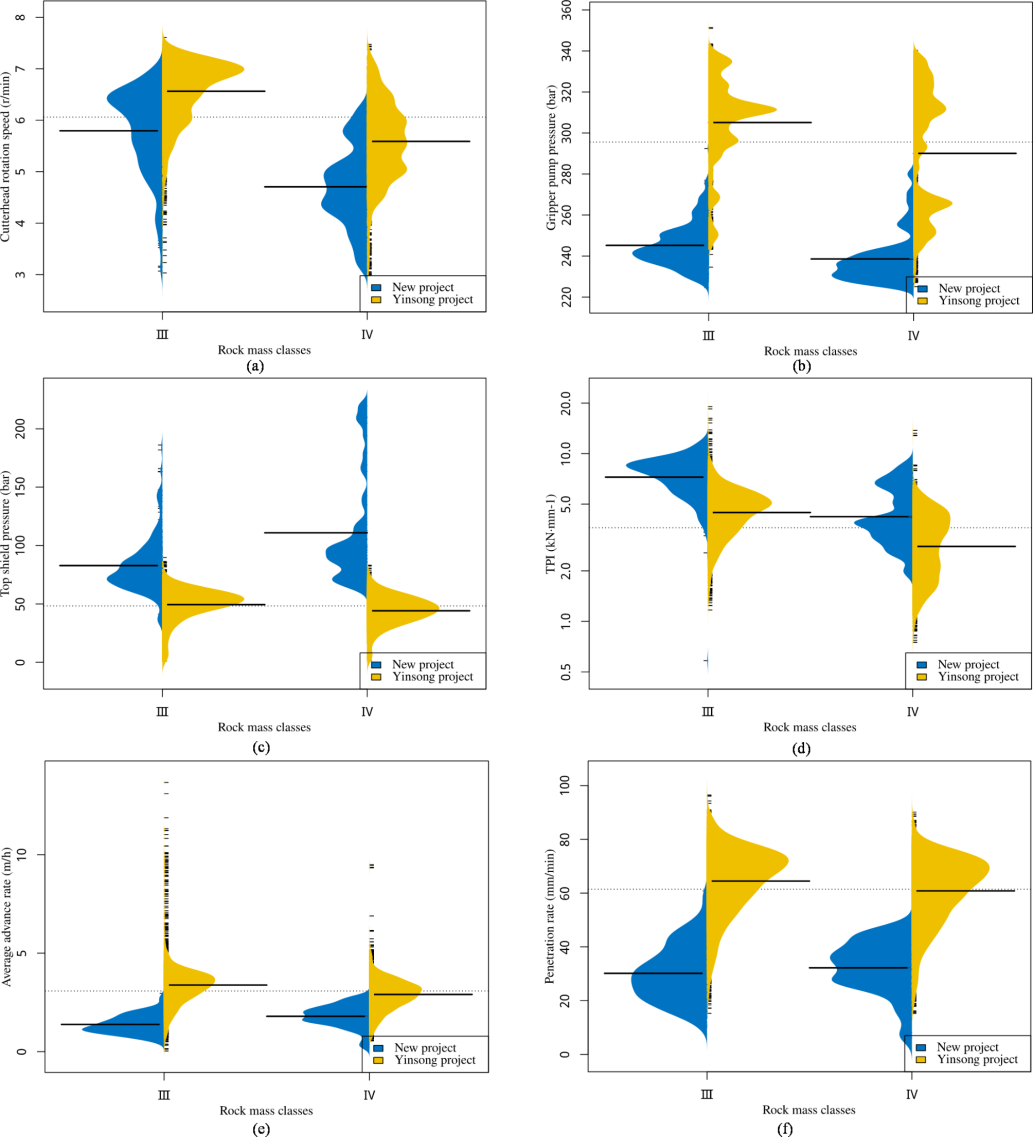
Bean plot analysis of public features: comparing Yinsong and new projects.

We evaluated the applicability of the model across various projects by examining the data distribution of common features in new projects. This comparison was made against the balanced engineering data of the Yinsong project, utilizing a bean plot. As a data visualization tool, the bean plot effectively illustrates the distribution characteristics and probability density of the data, offering an intuitive basis for comparing similar feature distributions across the two projects. Figure 14a–f show significant overlaps in the data distributions of the selected features between the two projects. This overlap indicates that despite differences in the projects, commonalities exist in certain key features, such as the tunnel diameters and rock mass classes, which jointly impact TBM construction. Next, the two parameters cutterhead rotational speed and TPI are taken as examples to elaborate.

The data analysis in Fig. 14a reveals a significant overlap in the cutter head rotational speed between the two projects in Classes III and IV. As a crucial parameter of TBM operation, the similar distribution of the cutterhead rotational speed across various projects suggests a consistent influence of factors such as the rock mass class on TBM construction, regardless of the differing project environments and conditions. This finding indicates that the TBM operational parameters, including the cutterhead rotational speed, can be effectively optimized based on experimental data from existing projects to enhance construction efficiency and safety.

The TPI, a crucial metric for assessing the performance of the TBM under individual disc cutters, reflects the efficiency and difficulty of the tunnel cutting process. In the analysis results shown in Fig. 14d, the TPI exhibits highly similar data distributions for both projects. This similarity suggests that when facing comparable geological conditions, different projects could consider adopting similar TBM configurations and operational strategies. This approach can potentially streamline project planning and execution by applying proven methods across similar geological contexts, thereby enhancing the overall efficiency and predictability of TBM-driven projects.

## Discussion

### Comparative analysis of SMOTE-based algorithms

In this study, we explored the improvement in the prediction performance of a rock mass classification model by implementing the MCT-RW algorithm. To assess the influence of the SMOTE-based algorithm on rock mass classification prediction using LightGBM, several variants were selected for comparative analysis, including SMOTE^29^, ADASYN^31^, borderline-SMOTE^32^, SMOTE-ENN^33^, *k*-means-SMOTE^34^, and Random-SMOTE^35^. The SMOTE-based algorithms generate new samples through linear interpolation between minority class samples and their nearest neighbors. Therefore, we investigated how the number of neighbors (*k*-value) in the SMOTE algorithm affects the prediction of rock mass classifications.

**Fig. 15:**
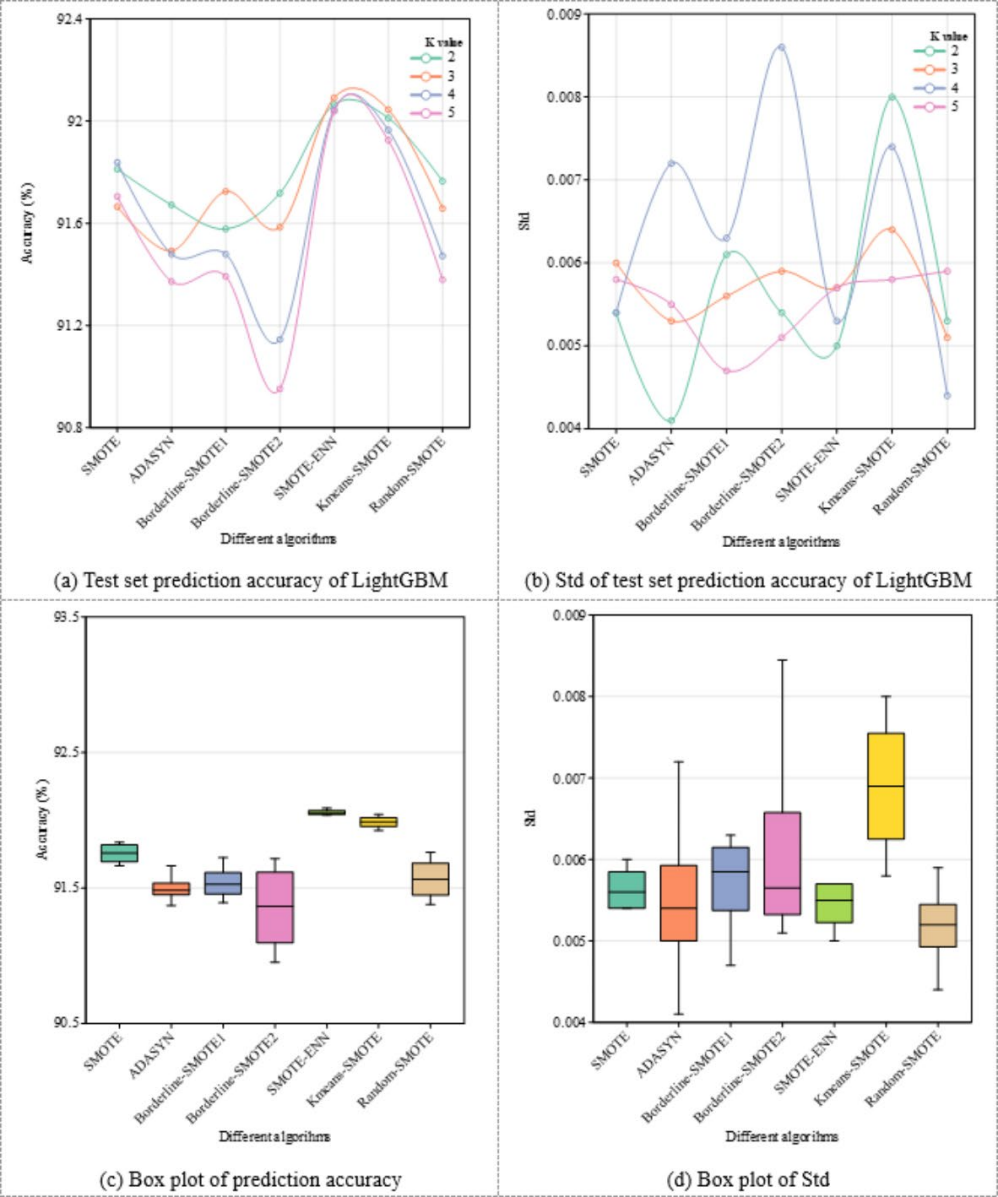
Comparative analysis of LightGBM test set predictions: accuracy and Std across SMOTE-based algorithm balanced data.

In Fig. 15a,c, the SMOTE-ENN and *k*-means-SMOTE algorithms with LightGBM maintain considerable stability in their performances across various *k*-values. Notably, their predictive outcomes are significantly superior to those of the models trained solely on the original dataset. Notably, an increase in *k* is associated with a reduction in the performance efficacy of most variants derived from the SMOTE algorithm. In particular, when algorithms such as SMOTE, ADASYN, and borderline-SMOTE with LightGBM were employed to balance the data in the rock mass classification, these approaches appeared to affect the overall effectiveness of the model inversely.

Figure 15b,d reveal that the SMOTE-ENN and *k*-means-SMOTE algorithms with LightGBM exhibit considerable stability in their performances across different *k*-values. Importantly, their predictive results significantly surpass those of models trained on the original dataset. However, an increase in *k* tends to diminish the performance of most SMOTE algorithm variants. In particular, when algorithms such as SMOTE, ADASYN, and borderline-SMOTE with LightGBM are used to balance the data in rock mass classification, these methods appear to affect the overall performance of the model inversely.

Considering both accuracy and Std, the SMOTE-ENN algorithm with LightGBM demonstrates better adaptability to balanced rock mass class data. This improvement could be due to the combination of the oversampling capability of SMOTE and the cleaning mechanism of ENN in the SMOTE-ENN algorithm. Smote not only increases the number of minority class samples, but also removes those that could lead to ambiguous classification decision boundaries. Consequently, the SMOTE-ENN algorithm with LightGBM, while improving the data balance, enhances the generalization ability and stability of the model. This makes it particularly effective for handling balanced rock-mass class data, offering a robust approach for dealing with imbalanced datasets in various contexts.

In contrast to SMOTE-based algorithms, the MCT-RW algorithm uses a comprehensive integration of statistical attributes and distance metrics to generate new samples. The samples generated by the MCT-RW algorithm closely align with the authentic distribution in the original dataset. This alignment makes it a suitable choice for improving model performance when used in conjunction with LightGBM.

### Comparative analysis of different algorithms

To delve deeper into the performance differences of various machine learning models in handling rock mass class data, both before and after data balancing, we carefully selected several classic machine learning algorithms for comparison, These algorithms include the random forest^52^, SVM^53^, *k*-nearest neighbor, and adaptive boosting^54^. To enhance the performance of the selected models and attain their optimal state, the IEWOA was employed to adjust and optimize the key hyperparameters.

In the random forest model, the maximum depth parameter controls the depth of the trees. It balances the model’s capacity to learn complex data patterns while preventing overfitting. A range of (1, 50] was selected to keep the model expressive without becoming overly complex. The number of estimators determines the total number of trees in the ensemble. This parameter directly affects model stability and predictive refinement. Setting it within the range of (1, 500] helps balance predictive accuracy with computational efficiency, avoiding diminishing returns. Minimum samples required for splits and leaf nodes manage the granularity of decision rules. They reduce the risk of overfitting by ensuring the model does not become overly tailored to the training data. These parameters are optimized within (1, 20] to enhance generalizability. For the SVM model, the RBF kernel was chosen for its effectiveness in capturing the non-linear relationships inherent in geological data. These relationships are critical for accurate classification. The regularization parameter (C) adjusts the balance between margin maximization and classification error minimization, enhancing model flexibility and robustness within the range of (1, 20]. The gamma parameter fine-tunes the influence of individual data points. The selected range of [0.001, 0.1] optimizes the model’s sensitivity to input features, ensuring an appropriate level of responsiveness. In the k-nearest neighbor algorithm, the number of neighbors controls the model’s responsiveness to local data variations. The range [2, 5] was selected to maintain stability without excessive sensitivity to noise. For adaptive boosting, the number of estimators determines the iterative boosting process, enhancing error correction capabilities. The learning rate adjusts the impact of each boosting iteration on the overall model. The ranges of (1, 500] and [0.001, 0.5] were chosen to balance rapid model adaptation and precise prediction refinement. The specific optimization ranges and explanations for these adjustments are presented in Table 6.

**Table 6:** Hyperparameters of random forest, SVM, and *k*-nearest neighbor and adaptive boosting optimization for rock mass class prediction.

	Hyperparameter	Explanation	Values	Type
Random forest	Maximum depth	Maximum depth of the tree	(1, 50]	Integer
	Number of estimators	Number of trees in the forest	(1, 500]	Integer
	Minimum number of samples when split	Minimum number of samples required to split an internal node	(1, 20]	Integer
	Minimum number of samples per leaf	Minimum number of samples required to be at a leaf node	(1, 20]	Integer
SVM	Kernel	Kernel type to be used in the algorithm	RBF	Categorical
	C	Regularization parameter	(1, 20]	Continuous
	Gamma	Kernel coefficient for RBF	[0.001, 0.1]	Continuous
*k*-nearest neighbor	Number of neighbors	Number of neighbors to use by default for k neighbors queries	[2, 5]	Integer
Adaptive boosting	Number of estimators	Maximum number of estimators at which boosting is terminated	(1, 500]	Integer
	Learning rate	Weight applied to each classifier at each boosting iteration	[0.001, 0.5]	Continuous

**Fig. 16:**
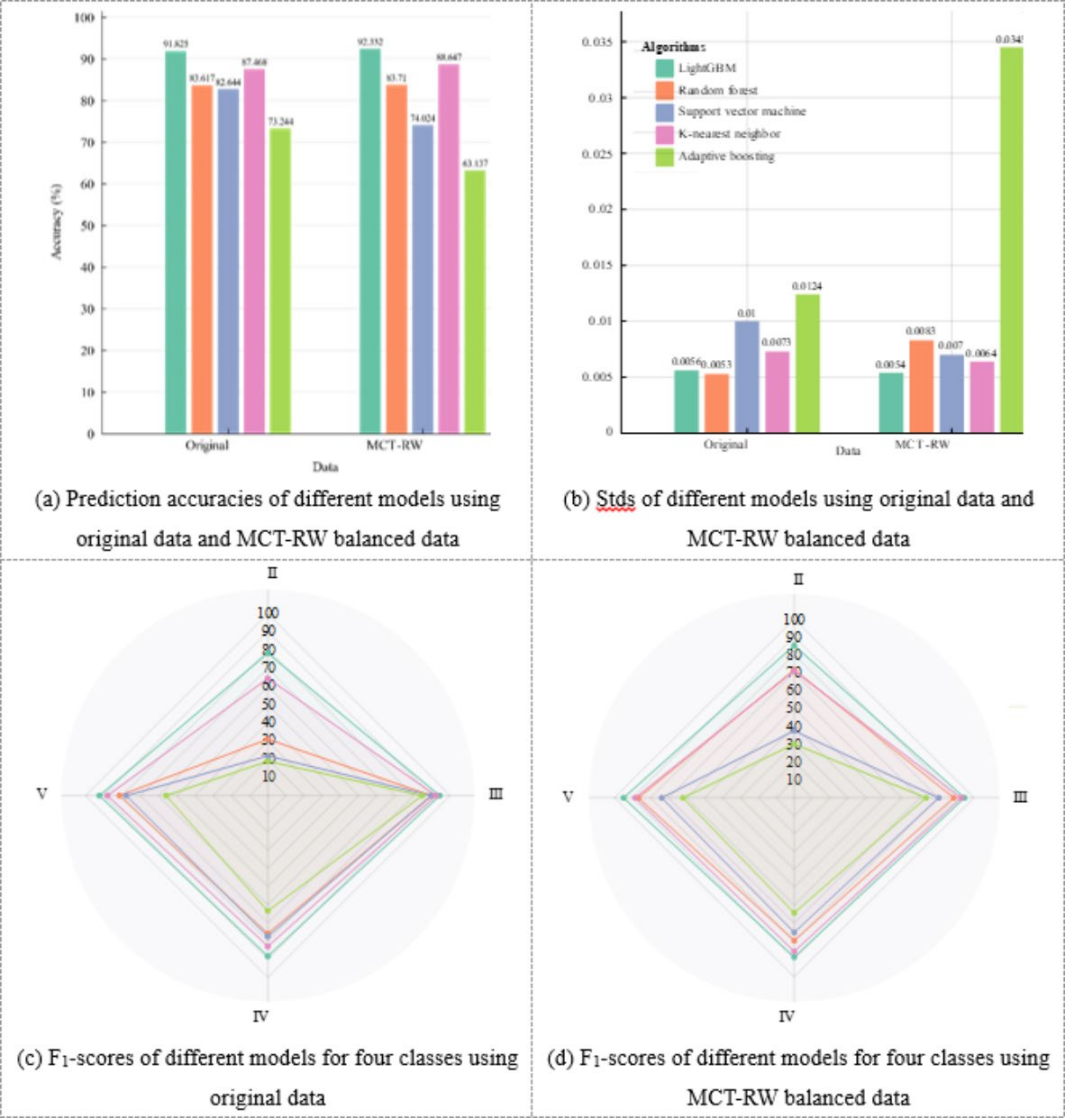
Accuracy, Std, and F_1_-score of LightGBM trained by original data and MCT-RW balanced data.

This assessment explored how different machine learning algorithms vary in the processing of rock mass class data. LightGBM demonstrates a remarkable improvement in accuracy, as shown in Fig. 16, with an increase from 91.825 to 92.332% after data balancing. This result highlights its adeptness in managing complex data features and efficiency in gradient boosting. The *k*-nearest neighbor algorithm also exhibits enhanced stability and accuracy, increasing from 87.468 to 88.647%, benefitting from the more balanced data that align well with its nearest neighbor voting mechanism. The random forest algorithm shows a modest improvement in accuracy from 83.617 to 83.71% post-data balancing, indicating a refined understanding of rock mass class features. Nonetheless, the shift in performance stability, with the Std changing from 0.0053 to 0.0083, indicates its sensitivity to altered data distribution patterns. Conversely, the SVM experiences a decrease in accuracy from 82.644 to 74.024%. This decline underlines its vulnerability to imbalanced datasets and sensitivity to data features, which likely affected its hyperplane optimization.

Adaptive boosting exhibits the most pronounced variance among the algorithms, with the accuracy decreasing from 73.244 to 63.137% after data balancing. The performance of the algorithm shows high variability, as reflected by the increase in the Std from 0.0124 to 0.0345. This result may be attributed to its reliance on weak learners, who are less effective with newly distributed data. These observations highlight the importance of algorithms like LightGBM and the *k*-nearest neighbor algorithm. These algorithms demonstrate strong adaptability to complex data variations, which is crucial for achieving precise rock mass classification in tunneling projects.

To facilitate a comprehensive comparison of different models for predicting rock mass classes, we adopted an evaluation approach based on the F_1_-score. This metric is particularly suitable for assessing the performance on imbalanced datasets, as it jointly considers precision and recall. The results presented in Fig. 16c indicate that LightGBM consistently achieves the highest F_1_-scores across all four classes.

LightGBM and the k-nearest neighbor algorithm stand out for their notably superior F_1_-score performance in Class II, IV, and V rock masses. Their performance is better compared to other models, including the random forest, SVM, and adaptive boosting algorithms. This distinction can be linked to the tailored effectiveness of LightGBM for rock mass class classification. It also reflects the efficiency of the *k*-nearest neighbor approach in processing datasets with clear feature distinctions. In the case of Class III, all models except adaptive boosting demonstrate similar predictive capabilities. The uniformity of Class III predictions may result from its dominance in the original dataset, which often leads to the misidentification of other rock mass classes as Class III. This trend highlights the intricacies involved in accurately classifying minority rock mass classes.

Figure 16d shows the F_1_-score dominance of LightGBM across all rock mass classes after data balancing, highlighting its adaptability. The performance of the random forest and *k*-nearest neighbor approaches also improves notably with balanced datasets. The SVM and adaptive boosting algorithms show improved performance in predicting Class II, reflecting their better identification of previously underrepresented classes. However, their performance decreases for Classes III, IV, and V, as they struggle to adapt to the new feature distribution. This result highlights the challenges faced by these models in adapting to data distribution changes. These trends underscore the varied applicability of data balancing across different models, revealing the complexity of optimizing machine-learning algorithms for diverse data scenarios.

### Effect of reducing rock mass classes from four to two on model performance

In Sect. 5.2, the limitations of the models in classifying the four rock mass classes were highlighted. To address these limitations, we developed a new classification strategy: merging Class II and Class III rock masses into “Class New II” and combining Class IV and Class V into “Class New IV.” This approach, based on tunneling parameter analysis, reveals similarities in the tunneling parameters for Classes II and III and similar data characteristics for Classes IV and V^20^. The objective of reclassification was to reduce confusion in finely divided classes, thereby enhancing the overall accuracy of model classification. Class New II comprised 4750 samples (63%), and Class New IV contained 2755 samples (37%). Models such as WOA-Original, WOA-MCT, WOA-RW, and their IEWOA-enhanced versions with LightGBM were trained and tested based on modified rock mass labels.

**Fig. 17:**
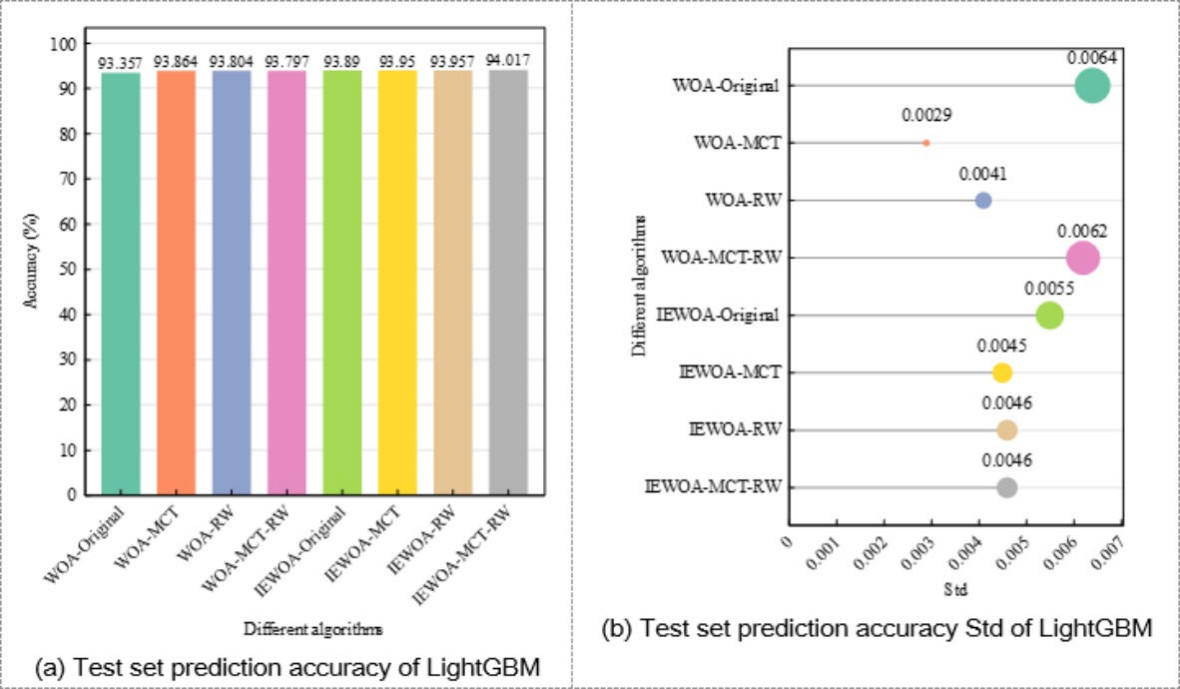
Accuracy and Std of LightGBM when reducing the number of rock mass classes from four to two.

The results shown in Figs. 10 and 17 consistently indicate that merging the four classes into two significantly enhances the model performance. For the initial four-class system, the model accuracies are as follows: WOA-Original, 91.339%; WOA-MCT, 91.772%; WOA-RW, 91.772%; WOA-MCT-RW, 92.139%; IEWOA-Original, 91.825%; IEWOA-MCT, 92.219%; IEWOA-RW, 92.219%; and IEWOA-MCT-RW, 92.332% with Stds ranging from 0.0042 to 0.0077. Upon consolidating these classes into two, the performance improves across all the models. The accuracies increase to 93.357% for WOA-Original, 93.864% for WOA-MCT, and 94.017% for IEWOA-MCT-RW. This change not only improves the accuracy by approximately 2%, but also results in more stable model behavior, evident in the reduced Std values.

**Fig. 18:**
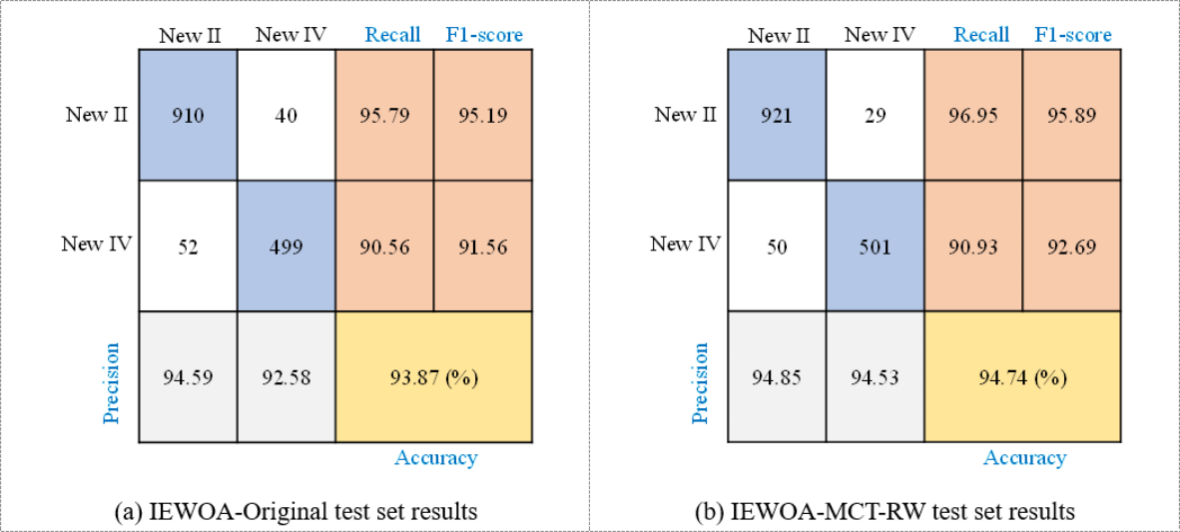
Confusion matrices of test set prediction of LightGBM when reducing rock mass classes from four to two.

The results in Fig. 18 demonstrate that the prediction performance of the test set using the IEWOA-MCT-RW algorithm with LightGBM significantly surpasses the results based on training with the original data. The IEWOA-MCT-RW algorithm with LightGBM achieves an accuracy of 94.74% and notably improves the recall rate for Class New II and precision for Class New IV. These results demonstrate that this approach is a more precise method of assessing the classes of rock masses that TBMs may encounter during tunnel construction.

This is particularly critical considering the physical characteristics of Class IV and V. Due to their softer nature compared to the other classes, the risk of collapse when a TBM operates in these rock masses is higher, as mentioned in^55^. Therefore, accurately and swiftly determining the class of rock masses in which a TBM operates is vital. It is essential for establishing appropriate construction strategies and ensuring the safety of TBM operations. The improved accuracy and reliability offered by the IEWOA-MCT-RW algorithm with LightGBM can be instrumental in making these crucial decisions.

The improved accuracy and superior performance metrics of the IEWOA-MCT-RW algorithm with LightGBM can be attributed to two principal enhancements: the integration of the IEWOA and MCT-RW components. The IEWOA introduces refined optimization strategies, encompassing advancements such as a modified parameter *l* and an innovative search strategy. Concurrently, the MCT-RW component pioneers a unique methodology for sample replication coupled with a sophisticated RW strategy. These synergistic improvements significantly augment the capability of the algorithm to discern swiftly and accurately the rock mass classes encountered by TBMs.

To further validate the performance of the proposed models, we applied IEWOA optimization and MCT-RW balancing on the newly merged classification dataset (Class New II and Class New IV), followed by training random forest, SVM, *k*-nearest neighbor, adaptive boosting, and XGBoost models for comparative analysis. As shown in Table 7, random Forest achieved an accuracy of 87.21%, SVM reached 88.01%, and *k*-nearest neighbor attained 92.94%, while adaptive boosting performed relatively poorly with an accuracy of only 74.95%. XGBoost achieved an accuracy of 93.80%, and LightGBM outperformed all other models with the highest accuracy of 94.74%. These results indicate that the optimization and balancing strategies of IEWOA and MCT-RW significantly enhance classification performance, with LightGBM and XGBoost outperforming other models, particularly LightGBM, which demonstrated the best performance across all metrics, highlighting its notable advantage.

**Table 7:** Performance comparison of models optimized with IEWOA and balanced with MCT-RW on the reclassified data set.

Models	Random Forest	SVM	k-nearest neighbor	Adaptive boosting	XGBoost	LightGBM
Accuracy	87.21%	88.01%	92.94%	74.95%	93.80%	94.74%

### Input feature sensitivity analysis

To investigate the sensitivity of features and the impact of input features on rock mass classification predictions, the SHapley Additive exPlanations^51^ (SHAP) library was employed as an interpretive tool^20^. SHAP is a widely used method for explaining the outputs of machine learning models, particularly useful for analyzing the influence of complex tunneling parameters on rock mass classification models. The underlying principle of SHAP is based on Shapley values from cooperative game theory, treating each tunneling parameter as a “player” contributing to the model’s decision-making process. By calculating the marginal contribution of each parameter across various combinations, SHAP determines the average impact of each feature on the predictive outcome. In our rock mass classification model, the absolute value of SHAP represents the importance of each tunneling parameter to classification accuracy, regardless of whether the effect is positive or negative; a higher absolute value indicates a greater impact of the parameter on the decision-making process. This approach helps identify the key parameters that significantly affect tunneling efficiency and classification accuracy, such as cutterhead rotation speed and gripper pressure, thereby enhancing the transparency of the model’s discrimination mechanism across different rock mass classes and providing data-driven support for optimizing parameter settings.

**Fig. 19:**
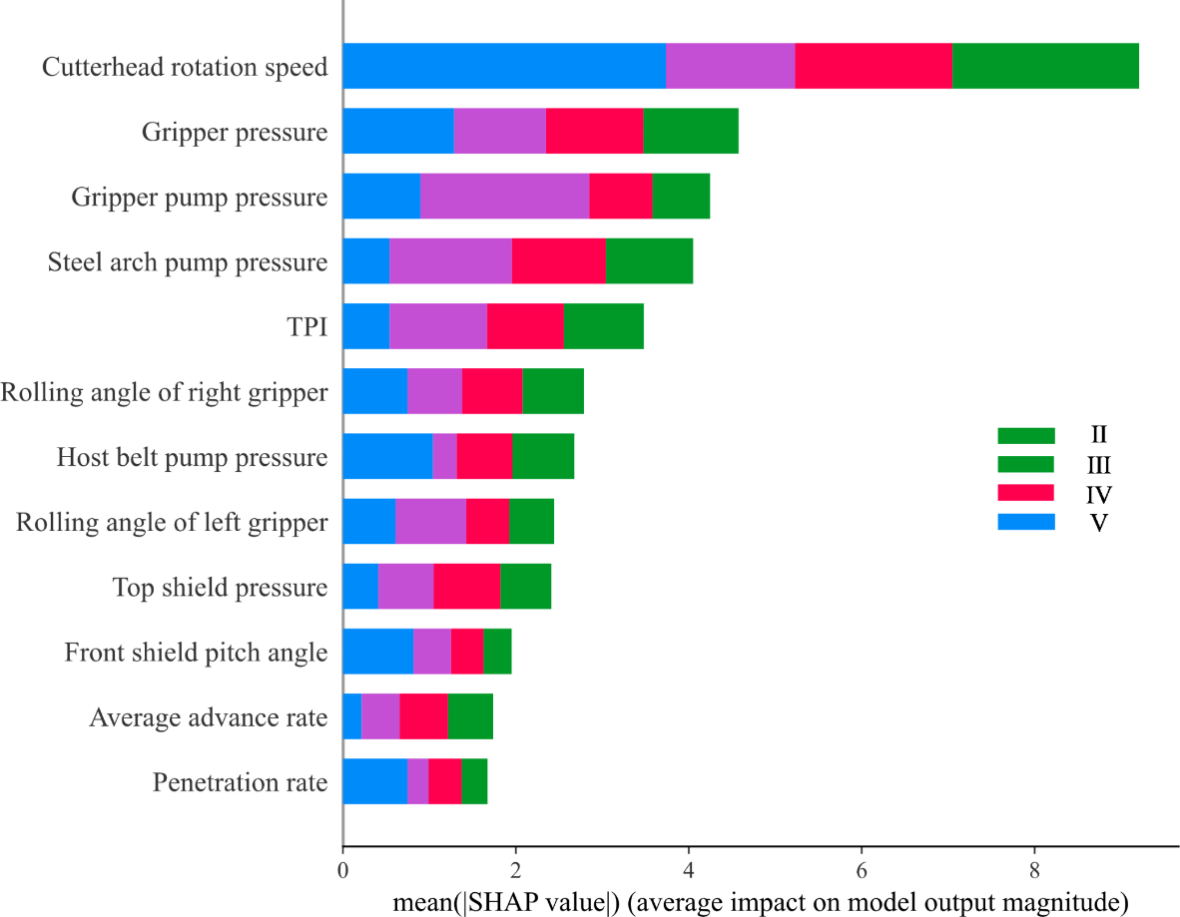
SHAP value analysis of tunneling parameters on rock mass classification sensitivity.

From the SHAP analysis shown in the Fig. 19, the absolute SHAP values of each tunneling parameter illustrate the sensitivity and influence of these features on the model’s output. Firstly, cutterhead rotation speed emerges as the most sensitive feature, with the highest SHAP absolute value, especially prominent in Class II, indicating that this parameter exerts the greatest influence on the model’s decision and is a critical control factor during the tunneling process. Following this, gripper pressure and gripper pump pressure exhibit substantial impacts in Classes II and III, demonstrating that these pressure control parameters are particularly vital under relatively stable rock mass conditions, significantly contributing to tunneling stability.

Steel arch pump pressure shows high sensitivity in Class III, highlighting the importance of this parameter in adjusting the support system for complex rock masses. TPI (thrust per inch) also contributes significantly to Class III, indicating its crucial role in optimizing tunneling speed. The rolling angle of right gripper and rolling angle of left gripper notably affect Classes III and IV, suggesting that adjusting gripper angles significantly enhances adaptability to varying rock masses. Additionally, host belt pump pressure and top shield pressure have substantial contributions in Class IV, emphasizing the importance of these pressures in supporting and regulating weaker rock masses. Front shield pitch angle shows a significant influence in Classes IV and V, underscoring the importance of shield angle adjustments in correcting tunneling paths. Finally, average advance rate and penetration rate display relatively balanced impacts across all classes but are slightly more sensitive in weaker rock masses (Class V), reflecting the necessity of controlling speed to ensure tunneling safety and efficiency. The SHAP analysis of these tunneling parameters reveals their respective impact patterns and significance in the model’s classification decisions. The sensitivity of different features to various rock mass classes provides clear guidance for optimizing tunneling strategies, offering scientific evidence for parameter adjustments under different rock mass conditions.

### Limitations and future work

A significant limitation of this study is its focus on validation projects with similar cutterhead diameters. This focus may not fully capture the variability in tunnel diameters and their corresponding tunneling parameters in various projects. The narrow scope restricts the applicability of the study results to a broader range of tunneling scenarios that involve different cutterhead sizes.

Another notable limitation is the study’s reliance on specific geological conditions and rock mass classes. These conditions may not adequately represent the diverse geological environments encountered in various tunneling projects. This specialization limits the applicability of the developed predictive models and algorithms. They may not effectively address the challenges posed by TBMs in different geological settings. Factors such as rock hardness, fracture patterns, and moisture content can significantly impact TBM performance; however, these factors were not fully explored in this study.

The study’s hyperparameter selection focused on key parameters identified as most influential based on prior research and empirical analysis. While this approach was effective, it did not explore all possible combinations. Consequently, some configurations that could improve performance under different conditions may have been overlooked. Future work should consider a broader range of hyperparameter settings to better understand their impact on model performance and stability in various tunneling scenarios.

The IEWOA-MCT-RW-LightGBM model achieves high accuracy but faces challenges in interpretability due to its hybrid structure. This complexity makes it difficult to identify the specific contributions of each component, potentially limiting its practical deployment where transparency is essential. Additionally, data-balancing methods like MCT-RW, while improving minority class representation, may alter the natural data distribution. This alteration could lead to overfitting or biased outcomes in different real-world scenarios.

Future research will aim to overcome these limitations by expanding the dataset to include a more diverse range of engineering projects. This expansion would improve the predictive accuracy and generalizability of the models across different tunneling scenarios, including varying tunnel diameters and geological conditions. Additionally, exploring a broader set of hyperparameters will help refine the models. It will provide deeper insights into optimizing TBM performance under different operational and environmental conditions.

## Conclusion

1. The IEWOA and its variant IEWOA-MCT-RW demonstrate superior performance across multiple benchmarks, achieving faster convergence rates and enhanced predictive capabilities compared to other algorithms, including WOA. The integration with LightGBM further enhances the model’s performance, particularly in predicting Classes II, IV, and V, where data balancing significantly improves recall and F_1_-scores.
2. The LightGBM model, trained on balanced data from the Yinsong project, improves the prediction accuracy of rock mass classes in new tunnel construction to 75.5%, outperforming the model trained on the original dataset (72.6%). This improvement is especially evident in Classes III and IV, indicating the benefits of data balancing. Analysis of data distribution highlights consistent key factors, such as cutterhead rotational speed and TPI, underscoring the model’s adaptability to new projects.
3. Among SMOTE-based methods, SMOTE-ENN and k-means-SMOTE exhibit superior stability, though increasing the k-value can negatively impact performance. SMOTE-ENN effectively manages balanced datasets through combined oversampling and cleaning mechanisms. In contrast, MCT-RW produces samples that accurately reflect the original data distribution by incorporating statistical attributes and distance metrics, offering a more precise replication than standard SMOTE variants.
4. In rock mass classification, LightGBM stands out due to its gradient boosting capability and resilience across varied data distributions. K-nearest neighbor excels in balanced datasets, but random forest, SVM, and adaptive boosting exhibit reduced stability post-balancing, revealing their limitations in adapting to data shifts. LightGBM’s adaptability and precision in complex scenarios affirm its superior role in rock-mass classification.
5. Reclassifying rock masses into Class New II and Class New IV based on tunneling parameters improved overall accuracy by 2% and enhanced model stability. The IEWOA-MCT-RW algorithm achieved 94.74% accuracy, outperforming traditional models through advanced optimization and sample replication techniques, validating the effectiveness of the proposed approach.

## Data Availability

The datasets generated and analyzed during the current study are not publicly available due requirements of our partners, but are available from the corresponding author on reasonable request.
